# Experimental validation of cuproptosis-associated molecular signatures and their immunological implications in pulmonary tuberculosis

**DOI:** 10.3389/fimmu.2025.1570992

**Published:** 2025-07-30

**Authors:** Xiaofang Liu, Qianqian Ma, Zhiming Li, Yong Xue, Jie Mi, Yuxi Li, Chunfeng Bai, Donglin Guo, Yinping Liu, Yan Liang, Jianqin Liang, Xueqiong Wu

**Affiliations:** ^1^ Beijing Key Laboratory of New Techniques of Tuberculosis Diagnosis and Treatment, Institute of Tuberculosis Research, Senior Department of Tuberculosis, the Eighth Medical Center of People’s Liberation Army (PLA) General Hospital, Beijing, China; ^2^ People's Liberation Army (PLA) General Hospital, Beijing, China; ^3^ Senior Department of Tuberculosis, The Eighth Medical Center of People's Liberation Army (PLA) General Hospital, Beijing, China

**Keywords:** pulmonary tuberculosis, cuproptosis, gene expression analysis, immune dysregulation, experimental validation

## Abstract

**Background:**

The pathogenic mechanism underlying *Mycobacterium tuberculosis* (MTB) remains elusive, posing challenges to its diagnosis and treatment. Cuproptosis is a newly identified mechanism of cell death. This study explores the role of cuproptosis-related genes (CRGs) in pulmonary tuberculosis (PTB) to uncover potential diagnostic biomarkers and therapeutic targets.

**Methods:**

Differentially expressed gene (DEG) analysis and weighted gene co-expression network analysis (WGCNA) were carried out using the GSE83456 dataset. PTB-associated DEGs were intersected with CRGs to identify PTB-related CRGs. Subsequent analyses included functional enrichment, gene interaction, and protein-protein interaction (PPI) network construction. Hub CRGs were screened out via least absolute shrinkage and selection operator (LASSO) regression and random forest (RF) algorithms. Diagnostic models were subsequently constructed and validated. The associations of immune cell infiltration and pathway with the identified hub genes were evaluated through single-sample gene set enrichment analysis (ssGSEA) and CIBERSORT. Hub gene expressions were validated in the GSE42834 and GSE89403 datasets, as well as by RT-qPCR and Western blot (WB) in PTB and extrapulmonary tuberculosis (EPTB) patients. The GSE89403 dataset and gene expression profiling were leveraged to analyze the differential expression of hub genes and their dynamic changes during treatment.

**Results:**

Seven PTB-related CRGs were significantly upregulated, were significantly upregulated, among which ASPHD2, GK, and GCH1 were identified as hub genes. These genes exhibited high expression levels in patients with PTB and EPTB, with marked reductions observed following treatment. Notable alterations in immune cell infiltration and immune function in PTB patients were closely related to these hub genes, suggesting activation of innate immune responses and suppression of adaptive immune function.

**Conclusion:**

The cuproptosis hub genes ASPHD2, GK, and GCH1 influence the pathogenesis of PTB, and possibly serve as novel diagnostic biomarkers and therapeutic targets.

## 1 Introduction

Tuberculosis (TB) is among the most severe infectious diseases worldwide. According to the World Health Organization (WHO) 2024 report, approximately 10.8 million new TB cases were reported globally in 2023, with nearly 1.25 million deaths. In addition, 400,000 new cases of multidrug-resistant and rifampicin-resistant TB were identified (1). With an estimated 741,000 newly diagnosed TB cases and approximately 29,000 new cases of multidrug-resistant or rifampicin-resistant TB (MDR/RR-TB) annually, China remains one of the countries bearing the highest global burden of both TB and drug-resistant TB (1). The overall TB control situation remains dire, as the Bacillus Calmette-Guérin (BCG) vaccine, the only currently licensed and widely administered TB vaccine, demonstrates limited efficacy in preventing adult TB. Early detection and effective management of drug-resistant TB remain formidable challenges due to the limited availability of efficacious anti-TB agents and the prolonged duration required for multidrug combination chemotherapy. Accordingly, elucidation of the pathogenic mechanisms of *Mycobacterium tuberculosis* (MTB) and the host’s protective immune responses may yield novel insights for the identification of diagnostic and therapeutic biomarkers, as well as the development of more effective vaccines and treatment strategies.

Recent studies have underscored the critical role of cell death pathways in modulating host resistance to MTB infection and facilitating immune evasion by the pathogen. Apoptosis, pyroptosis, and autophagy are recognized as protective mechanisms that restrict intracellular bacterial proliferation and enhance host immune defense against MTB. In contrast, necrosis and ferroptosis are generally associated with detrimental outcomes, as they promote MTB survival and dissemination (2). In 2022, Tsvetkov P et al. first proposed cellular cuproptosis, a novel form of regulated cell death triggered by excessive copper ions, which represents a distinct mechanism of cell death (3).

Copper, an essential trace element in the human body, is crucial as a catalytic cofactor in various biological processes and involved in many vital physiological functions. The body meticulously regulates copper absorption, distribution, and elimination to maintain low intracellular concentrations of free copper ions, thereby preserving cellular copper homeostasis and minimizing copper-induced cellular damage. In the event of intracellular copper dysregulation, excess copper ions bind to acylated mitochondrial enzymes involved in the copper-dependent tricarboxylic acid (TCA) cycle, leading to protein aggregation, proteotoxic stress, and ultimately the induction of cell death (3–7). The role of cuproptosis in TB remains an area of early investigation. Li et al. utilized the Gene Expression Omnibus (GEO) dataset GSE83456 to examine blood gene expression profiles from healthy individuals and patients with TB, identifying 11 differentially expressed cuproptosis-related genes (CRGs). Among TB patients, NFE2L2, NLRP3, ATP7B, SLC31A1, MTF1, and DLD were significantly upregulated, whereas LIAS, LIPT1, DLAT, GLS, and DBT were downregulated (8). In a separate study based on the GSE39939 dataset, Chen et al. investigated the expression patterns and immunological characteristics of copper metabolism regulatory genes in pediatric patients with active tuberculosis (ATB) and latent tuberculosis infection (LTBI). They identified nine differentially expressed CRGs associated with active immune responses. Specifically, MTF1, NFE2L2, and NLRP3 were upregulated, while FDX1, LIPT1, PDHB, GLS, DBT, and DLST were downregulated in the ATB group relative to the LTBI group. Moreover, two distinct molecular subtypes related to cuproptosis were delineated among children with ATB. Subtype 1 was characterized by reduced lymphocyte levels and heightened inflammatory activation compared to Subtype 2. Correspondingly, Subtype 1 exhibited increased expression of MTF1, NFE2L2, and NLRP3, whereas Subtype 2 demonstrated higher expression of LIPT1, PDHB, GLS, and DBT. Based on these findings, a predictive model incorporating five CRGs (MAN1C1, DKFZP434N035, SIRT4, BPGM, and APBA2) was constructed to differentiate children with ATB from those with LTBI. This model demonstrated robust predictive performance (9). Current evidence has demonstrated the important role of cuproptosis in the development of TB. These findings highlight the imperative for further exploration into the immunopathological mechanisms underpinning the cuproptosis pathway in pulmonary tuberculosis (PTB). Continued research, including experimental validation of novel biomarkers related to diagnosis, therapeutic response, and prognosis, holds considerable promise for clinical application.

This study, based on the GEO data, explores the differential expression, biological functions, and regulatory pathway alterations of CRGs between healthy individuals and patients with PTB. Machine learning (ML) algorithms were employed to identify PTB-associated hub CRGs, whose diagnostic potential was subsequently evaluated via receiver operating characteristic (ROC) curve analysis. A diagnostic model was constructed and validated accordingly. In addition, the immunological profiles of these hub genes were systematically examined. Validation of hub gene expression was conducted via the GEO datasets GSE42834 and GSE89403, and dynamic changes in their expression during treatment were further analyzed. For the first time, differential expression of these hub genes was assessed among healthy controls, PTB patients, and extrapulmonary tuberculosis (EPTB) patients using quantitative real-time reverse transcription polymerase chain reaction (RT-qPCR), Western blot (WB), and transcriptomic profiling. Moreover, dynamic expression patterns of these genes throughout clinical treatment were characterized, providing a potential foundation for the development of efficacy-monitoring strategies.

## 2 Materials and methods

**Figure 1:**
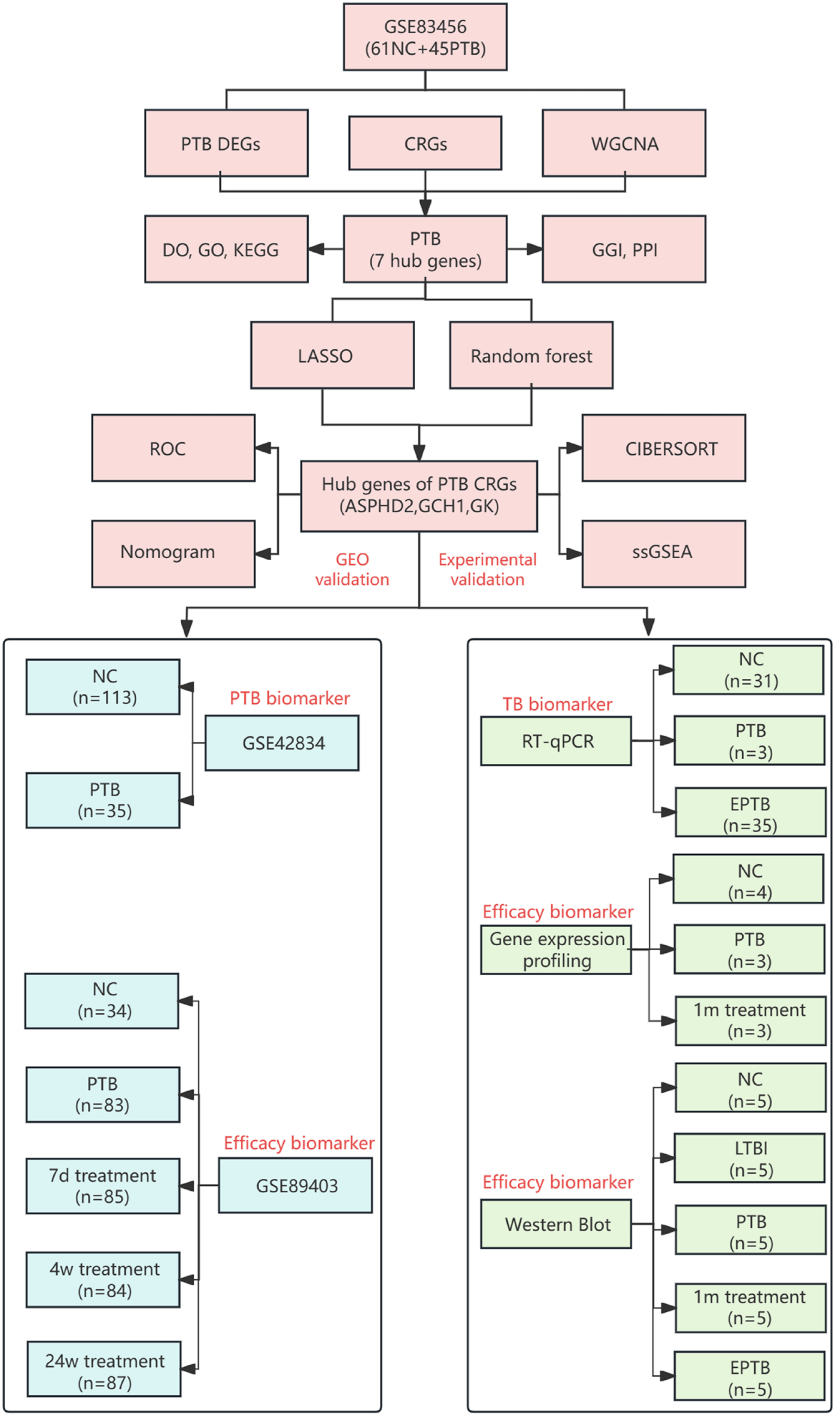
Flowchart of identification and experimental validation of key CRGs in TB. NC (normal control), PTB (pulmonary tuberculosis), LTBI(latent tuberculosis infection), EPTB (extrapulmonary tuberculosis), DEGs (differentially expressed genes), WGCNA (weighted gene co-expression network analysis), CRGs (cuproptosis-related genes), DO (disease ontology), GO (gene ontology), KEGG (Kyoto encyclopedia of genes and genomes), GGI (gene-gene interaction), PPI (protein-protein interaction), LASSO (least absolute shrinkage and selection operator), ROC (receiver operating characteristic curve), CIBERSORT (cell-type identification by estimating relative subsets of RNA transcripts), ssGSEA (single-sample gene set enrichment analysis), GEO (gene expression omnibus), PCR (polymerase chain reaction), 1m treatment (1-month treatment), 7d treatment (7-day treatment), 4w treatment (4-week treatment), and 24w treatment (24-week treatment).

The research process is illustrated in **Figure 1**.

### 2.1 Data downloading and pre-processing

The gene expression profiles for PTB were retrieved from the GEO (http://www.ncbi.nlm.nih.gov/geo/). Transcriptomic datasets were retrieved from GEO using the terms: “pulmonary tuberculosis,” “active tuberculosis,” “Homo sapiens,” “whole blood,” “PBMCs,” “RNA-Seq,” “gene expression,” and “microarray.” Inclusion criteria were: (i) RNA expression data (microarray or RNA-Seq); (ii) samples from whole blood or PBMCs; (iii) datasets containing both PTB and healthy controls; (iv) publication within the past 15 years. Exclusion criteria included: (i) non-blood samples (e.g., lung tissue, sputum); (ii) non-expression data (e.g., methylation, proteomics, miRNA only); (iii) lack of group annotation or technical replicates with incomplete metadata; and (iv) for duplicate probes, the one with the highest mean expression was retained. Full dataset details are provided in **Table 1**. The dataset GSE83456 was selected, which includes 106 blood samples, comprising 61 normal control (NC) samples and 45 PTB samples. The GSE42834 and GSE89403 datasets were treated as validation sets. GSE42834 contains 35 PTB blood samples and 113 NC blood samples, while GSE89403 includes 34 NC blood samples and 83 PTB blood samples, with blood samples from PTB patients at 7 days, 4 weeks, and 24 weeks of treatment. The raw microarray data were primarily processed via R (v4.3.0). Platform-specific annotation files were used to convert probe IDs to gene symbols. For genes mapped by multiple probes, the one with the highest expression value was retained. Probes with missing values were removed. The expression matrices were transposed and subsequently merged with sample metadata. The Perl programming language was utilized solely for automating the batch mapping of probes to genes.

**Table 1:** Datasets used in the study.

Dataset ID	Year	Ethnicity	Study design	Platform	HC (n)	PTB (n)	Notes
GSE83456	2016	UK (Mixed)	Case-Control	Illumina Human HT-12 V4 BeadChip	61	45	None
GSE42834	2013	White, Black, ISC, Middle Eastern, SE Asian, and Central Asian	Case-Control	Illumina Human HT-12 V4 BeadChip	113	35	None
GSE89403	2017	South Africa (Mixed)	Case-Control	Illumina (San Diego, CA) HiSeq-2000	34/38	83/100	Excluded samples lacking clear clinical group annotation or containing technical replicates with incomplete metadata.

### 2.2 Weighted gene co-expression network analysis

WGCNA was conducted on the pre-processed GSE83456 dataset using the WGCNA software package. A soft-thresholding power of 6 was selected to ensure the construction of a scale-free network. Gene co-expression modules were identified through the dynamicTreeCut algorithm, and module eigengenes (MEs) were subsequently calculated. These MEs were correlated with clinical phenotypes to determine differentially expressed genes (DEGs) within the modules that showed significant associations. The R packages used in this step were WGCNA (v1.72-1), limma (v3.56.2), and dynamicTreeCut (v1.63-1).

### 2.3 DEG analysis and functional enrichment analysis

The “limma” package (v3.56.2) was used to identify DEGs between PTB patients and NC samples, with a selection criterion of |log2 FC| > 1 and adjusted p-value (FDR) < 0.05, corrected through the Benjamini-Hochberg method. 190 DEGs were extracted from PTB samples and subsequently used to identify those related to PTB via WGCNA. These DEGs were intersected with CRGs (3). Subsequently, “DOSE”(v3.26.1), “org.Hs.eg.db”(v3.17.0), and “clusterProfiler”(v4.8.1) packages were subsequently used for Disease Ontology (DO), Gene Ontology (GO), and Kyoto Encyclopedia of Genes and Genomes (KEGG) functional enrichment analyses to elucidate potential functions and pathways of cuproptosis-related DEGs in the pathogenesis of PTB.

### 2.4 Gene-gene and protein-protein interaction networks

Gene-gene interaction (GGI) data were obtained from the GeneMANIA database (http://genemania.org), and PPI data for intersecting genes were obtained from the STRING database (http://string-db.org). Interaction networks were constructed and visualized to analyze gene-protein relationships, thereby elucidating disease pathogenesis and potential therapeutic targets.

### 2.5 ML algorithms

Significant predictive genes were selected through the least absolute shrinkage and selection operator (LASSO) regression model (glmnet package v4.1-8) and the random forest (RF) algorithm (randomForest package v4.7-1.1). LASSO was employed to perform feature selection by penalizing and minimizing the absolute values of regression coefficients. In parallel, the RF algorithm assessed the relative importance of each gene through the construction of multiple decision trees. To ensure the robustness and generalizability of the results, a 10-fold cross-validation strategy was implemented for LASSO, while a train-test split approach was adopted for RF. Cross-validation serves to reduce the risk of overfitting and enhances model stability across varying data partitions. The outputs from both algorithms were subsequently integrated to identify the hub genes associated with cuproptosis in PTB.

### 2.6 Construction and validation of diagnostic predictive models

To assess the diagnostic value of cuproptosis hub genes in PTB, models were constructed and validated via the “rms” (v6.7-0), “stdca” (v1.0), “pROC” (v1.18.5), and “ggplot2” (v3.5.1) packages. Based on the GSE83456 dataset, a nomogram model incorporating ASPHD2, GK, and GCH1 genes was developed through the lrm() function from the rms package. The model’s prediction performance was rigorously rated through calibration curves and decision curve analysis (DCA). Additionally, the ROC curve and the corresponding area under the curve (AUC) were employed to evaluate the model’s discriminative ability.

### 2.7 CIBERSORT analysis of immune infiltration

The CIBERSORT algorithm was employed to analyze the GSE83456 dataset and to infer the relative proportions of 22 distinct immune cell subsets, to elucidate the influence of cuproptosis-related hub genes on immune cell infiltration in PTB. Only samples with a CIBERSORT-derived p-value less than 0.05 were retained for downstream analyses. Gene expression data were normalized utilizing the limma package (v3.56.2). Subsequently, expression matrices corresponding to the identified hub genes were extracted for further analysis. Spearman correlation analysis was performed to assess the relationships between hub gene expression levels and the relative abundance of immune cell populations. Data visualization was conducted employing the ggplot2 (v3.4.2), reshape2 (v1.4.4), and tidyverse (v2.0.0) packages. Quantile normalization and support vector regression required by the CIBERSORT procedure were implemented using preprocessCore (v1.62.1) and e1071 (v1.7-13).

### 2.8 Single-sample gene set enrichment analysis

The GSE83456 dataset was analyzed through ssGSEA to evaluate the expression levels of cuproptosis hub genes in single samples. Gene set enrichment scores for each sample were calculated using the “GSVA” package (v1.46.0) in R (v4.3.0), with gene sets parsed by GSEABase (v1.62.0). Gene expression data were normalized via the “limma” package (v3.56.2). Spearman correlation analysis was conducted to evaluate the association between gene expression levels and immune cell infiltration. The results were visualized employing “ggplot2” (v3.4.2), “reshape2” (v1.4.4), and “ggpubr” (v0.6.0).

### 2.9 Peripheral blood sample collection

Blood samples for RT-qPCR validation of cuproptosis hub genes in PTB were collected between March 2024 to December 2024. These samples were obtained from 31 healthy controls, 35 PTB patients, and 34 EPTB patients attending the Physical Examination Center and the Senior Department of Tuberculosis of the Eighth Medical Center of PLA General Hospital (**Table 2**). For gene chip validation, blood samples were also collected from four healthy controls and three PTB patients before and after one month of anti-TB treatment with rifampicin, isoniazid, pyrazinamide, and ethambutol (HRZE) regimen. For WB validation, five individuals were selected from each of the following groups: HC, LTBI, PTB, PTB patients after one month of anti-TB treatment (AT), and EPTB. The inclusion criteria were as follows:

1. NC group: (i) negative results of IFN-γ release assay; (ii) no abnormalities on chest CT examination.
2. PTB group: (i) clinical diagnosis of PTB according to the standards of *Diagnosis of Pulmonary Tuberculosis* (WS 288-2017) (10) issued by the National Health and Family Planning Commission of the People’s Republic of China; (ii) no history of anti-TB treatment, or anti-TB treatment with the HRZE regimen for less than one week.
3. EPTB group: (i) clinically diagnosed as EPTB according to the standards of the *Classification of Tuberculosis* (WS 196-2017) (11) published by the Infectious Disease Control Institute of the Chinese Center for Disease Control and Prevention.
4. LTBI group: Positive IFN-γ release assay (IGRA) results, indicating LTBI; No clinical signs of ATB, defined by negative radiology, sputum smear, and sputum culture for *MTB*.

**Table 2:** The demographics and clinical characteristics of patients with tuberculosis and normal controls.

Characteristic	PTB patients (*n*=35)	EPTB patients (*n*=34)	Normal controls (*n*=31)
Sex (female/male)	15/20	18/16	15/16
Age (years, mean ± SD)	43.92 ± 17.54	40.03± 16.09	39.39 ± 11.64
classification of diseases (yes/no)	Infiltrative Pulmonary Tuberculosis(25/10)	Lymph Node Tuberculosis(8/26)	NA
		Genitourinary Tuberculosis(14/20)	
	Cavitary Pulmonary Tuberculosis (9/26)	Gastrointestinal Tuberculosis(6/28)	
		Tuberculous Meningitis(10/24)	
	Tracheobronchial Tuberculosis (12/23)	Tuberculous Spinal Meningitis(3/31)	
		Tuberculosis of the Ear(1/33)	
	Tuberculous Pleurisy (10/25)	Osteoarticular Tuberculosis(7/27)	
		Abdominal Tuberculosis(3/31)	

Exclusion criteria were as follows: (i) severe liver or kidney disease; (ii) HIV infection or other autoimmune diseases; (iii) pregnancy; (iv) recent use of immunomodulators; (v) age < 18 or > 70 years. The research protocol was reviewed and approved by the Ethics Committee of the Eighth Medical Center of PLA General Hospital (approval number: 30920230831122735), and informed consent was obtained from all participants.

### 2.10 RT-qPCR

Peripheral blood mononuclear cells (PBMCs) were isolated from peripheral whole blood specimens via density gradient centrifugation employing Ficoll-Paque (GE Healthcare Life Sciences, USA). Subsequently, the isolated cells were stimulated with recombinant TB-CFP-10-ESAT-6 protein (Gene Optimal, Shanghai, China) at a concentration of 90 µg/mL for 24 hours. Total RNA was subsequently extracted utilizing TRIzol^®^ Reagent (Invitrogen, USA) as per the manufacturer’s protocol. The extracted RNA was subsequently reverse-transcribed into complementary DNA (cDNA) using the PrimeScript™ RT Reagent Kit (TaKaRa Biotechnology, Japan) (12, 13). Quantitative PCR (qPCR) amplification was performed on a QuantStudio 384 Gene Amplification System (Thermo Fisher Scientific, USA) with the following conditions: 2 minutes of pre-denaturation at 95°C, followed by 40 cycles (denaturation at 95°C for 1 second and annealing/extension at 60°C for 30 seconds) using a rapid cycling mode. GAPDH was used as an internal reference, and the relative expression of each gene was calculated via the 2−△△Ct method. The primer sequences for each gene are shown in **Table 3**.

**Table 3:** Primers sequence of target gene amplification.

Gene	Complete gene name	Forward (5′-3′)	Reverse (5′-3′)
GK	Glycerol Kinase	GAACCCAGTCTACCGTTGAGA	TGGACACCTCCATTGACTCCT
GCH1	GTP Cyclohydrolase 1	GTGAGCATCACTTGGTTCCAT	GTAAGGCGCTCCTGAACTTGT
ASPHD2	Aspartate Beta-Hydroxylase Domain Containing 2	CCGAGGACTGATTGTCTGACC	CAGTACCACACGAAGAGGACC
GAPDH	Glyceraldehyde-3-Phosphate Dehydrogenase	CTCTGGTAAAGTGGATATTGT	GGTGGAATCATATTGGAACA

### 2.11 Gene expression profiling analysis

Peripheral blood mononuclear cells (PBMCs) were isolated from peripheral whole blood samples. Total RNA was subsequently extracted utilizing TRIzol reagent and submitted to Shanghai Aksomics Co., Ltd. (China) for gene expression profiling. RNA purity and concentration were assessed with a NanoDrop ND-1000 spectrophotometer, exhibiting A260/A280 ratios ranging from 1.8 to 2.4 and A260/A230 ratios between 1.5 and 2.4, with an approximate RNA concentration of 100 ng/μL. RNA integrity was confirmed via denaturing gel electrophoresis. Gene expression analysis was performed using human whole-genome oligonucleotide microarrays encompassing annotated genes and transcripts. Following the Agilent one-color microarray-based gene expression protocol, total RNA was linearly amplified and labeled with Cy3-UTP. The resultant labeled complementary RNA (cRNA) was purified using the RNeasy Mini Kit. Hybridization was conducted on Agilent microarrays, followed by washing, fixation, and scanning of the arrays. Signal intensities were extracted using Agilent Feature Extraction software, and data normalization was performed employing the Quantile normalization method.

### 2.12 WB analysis

Peripheral blood mononuclear cells (PBMCs) were isolated from peripheral whole blood samples by density gradient centrifugation via Ficoll-Paque (GE Healthcare Life Sciences, USA). Cellular lysis was performed using radioimmunoprecipitation assay (RIPA) buffer (Beyotime, China) to extract total protein. Protein concentrations were quantified employing the bicinchoninic acid (BCA) Protein Assay Kit (Thermo Fisher Scientific, USA), and all samples were adjusted to a final concentration of 0.3 µg/µL.

Equal amounts of protein (6 µg) were separated by SDS-PAGE (12% gel, LabPAGE, LABLEAD, Beijing, China) and subsequently transferred to 0.45 µm nitrocellulose membranes (Merck Millipore, IRL). Membranes were subsequently blocked for 15 minutes at room temperature using Rapid Blocking Buffer (TBS-T) (LABLEAD, Beijing, China) containing 0.1% Tween-20.

Target proteins ASPHD2, GCH1, and GK were detected using specific primary antibodies: a mouse monoclonal antibody for ASPHD2, and rabbit polyclonal antibodies for GCH1 and GK. Internal loading controls comprised GAPDH, mouse-derived for ASPHD2 detection rabbit-derived for GK, and β-actin for GCH1. Primary antibodies were diluted as follows: ASPHD2 (1:1000), GCH1 (1:2500), GK (1:2500), and both GAPDH and β-actin (1:2500). Horseradish peroxidase (HRP)-conjugated secondary antibodies (Proteintech, China) were applied at a 1:4000 dilution and incubated with the membranes.

Protein signals were visualized using enhanced chemiluminescence (ECL) substrate (Thermo Fisher Scientific, USA) and captured with the AI600QC ultra-sensitive multifunctional imaging system (GE Healthcare, USA). Densitometric analysis of protein bands was performed using ImageJ software (National Institutes of Health, Bethesda, MD, USA). Normalized expression levels were statistically analyzed with GraphPad Prism.

### 2.13 Statistical methods

All analyses were enabled by R (v4.3.0). Statistical analyses were performed using GraphPad Prism 9 and IBM SPSS Statistics 25. Results for clinical general data were displayed as mean ± standard deviation (Mean ± SD), and a p-value below 0.05 denoted statistical significance. The normality of data distribution was evaluated using the Shapiro-Wilk test or the Kolmogorov-Smirnov test, as appropriate. For comparisons involving more than two groups, a one-way analysis of variance (ANOVA) was conducted to assess intergroup differences. In instances where the data did not conform to a normal distribution, the Kruskal-Wallis test was employed. For comparisons between two groups, the unpaired two-tailed Student’s t-test was applied to normally distributed data, whereas the Mann-Whitney U test was used for non-normally distributed data. WB data were analyzed by computing the effect size (Cohen’s d) using the cohen.d() function from the effsize package. Effect sizes were interpreted per conventional thresholds: small (d ≥ 0.2), medium (d ≥ 0.5), and large (d ≥ 0.8). *p* < 0.05 denoted statistical significance, and the specific statistical methods applied are indicated in the figure legends. Asterisks represent significance levels as follows: * *p* < 0.05, ** *p* < 0.01, *** *p* < 0.001.

## 3 Results

### 3.1 Identification of DEGs in PTB

**Figure 2:**
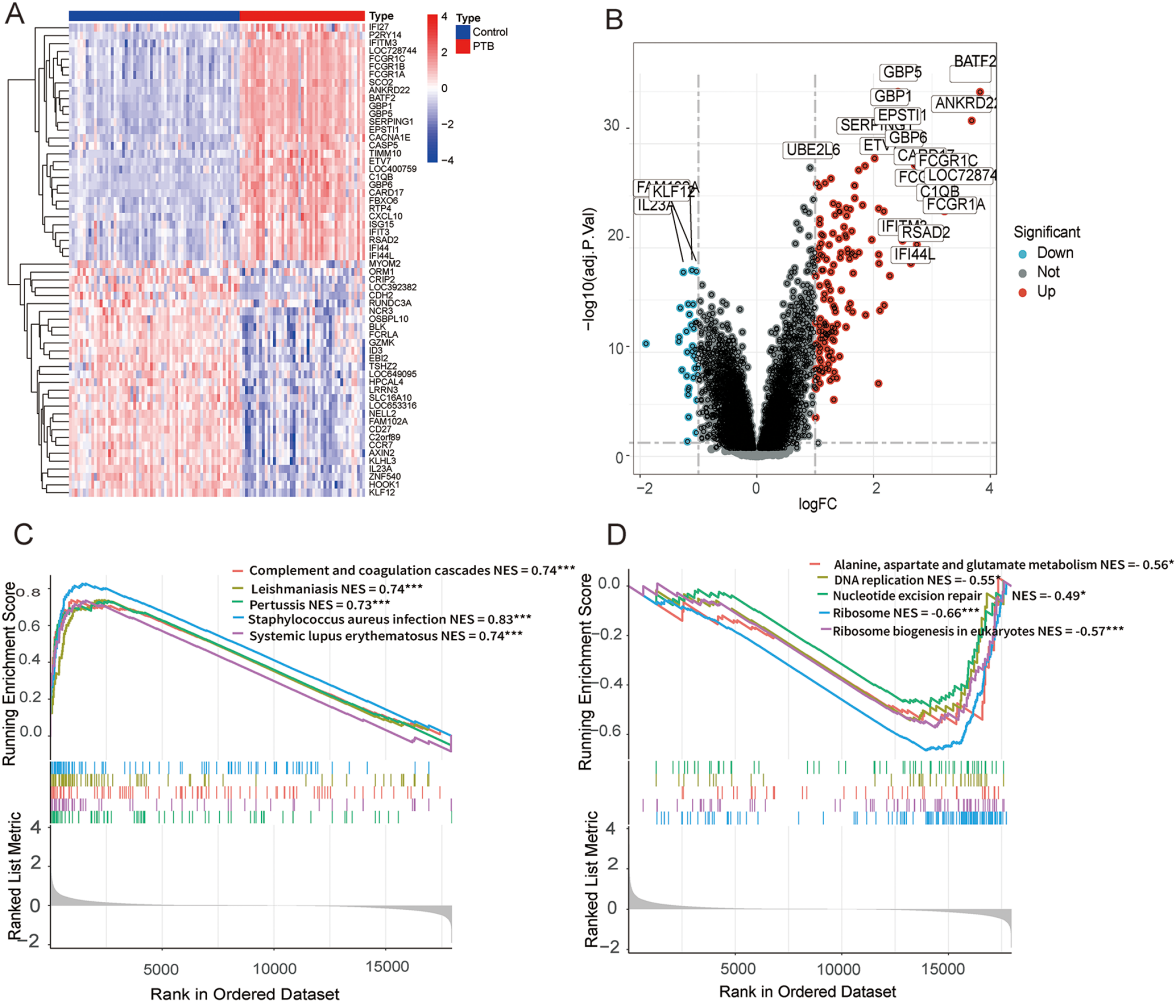
DEGs and functional enrichment analysis between PTB and normal samples in the GSE83456 dataset. **(A)** A microarray heatmap and hierarchical clustering of DEGs. Upregulated genes are displayed in red, while downregulated genes are shown in blue. **(B)** A volcano plot illustrating the DEGs between PTB and normal samples. The red dots represent upregulated DEGs, the green dots represent downregulated DEGs, and the black dots denote genes with no significant changes. **(C)** GSEA results show significant enrichment of upregulated genes in immune-related pathways, including Staphylococcus aureus infection (NES = 0.83, FDR < 0.001), Leishmaniasis (NES = 0.74, FDR < 0.001), Complement and coagulation cascades (NES = 0.74, FDR < 0.001), Systemic lupus erythematosus (NES = 0.74, FDR < 0.001), Pertussis (NES = 0.73, FDR < 0.001). **(D)** GSEA results show significant enrichment of downregulated genes in pathways related to ribosome and metabolism, including Ribosome (NES = -0.66, FDR < 0.001), Ribosome biogenesis in eukaryotes (NES = -0.57, FDR < 0.001), Alanine, aspartate and glutamate metabolism (NES = -0.56, FDR = 0.041), DNA replication (NES = -0.55, FDR = 0.047), Nucleotide excision repair (NES = -0.49, FDR = 0.039). *p < 0.05, **p < 0.01, ***p < 0.001.

In the GSE83456 dataset, 190 DEGs were identified with |log2 FC| > 1 and adjusted p-value (FDR) < 0.05 as screening criteria. Among them, 152 were significantly upregulated and 38 were significantly downregulated in PTB samples (**Figures 2A, B**).

To further elucidate the functional roles of these DEGs, gene set enrichment analysis (GSEA) was conducted on the entire gene set. The analysis revealed that the upregulated genes were predominantly enriched in pathways on immune responses and pathogen infection, whereas the downregulated genes were chiefly enriched in pathways involved in ribosome biogenesis and metabolic processes. (**Figures 2C, D**).

### 3.2 WGCNA analysis and module identification

**Figure 3:**
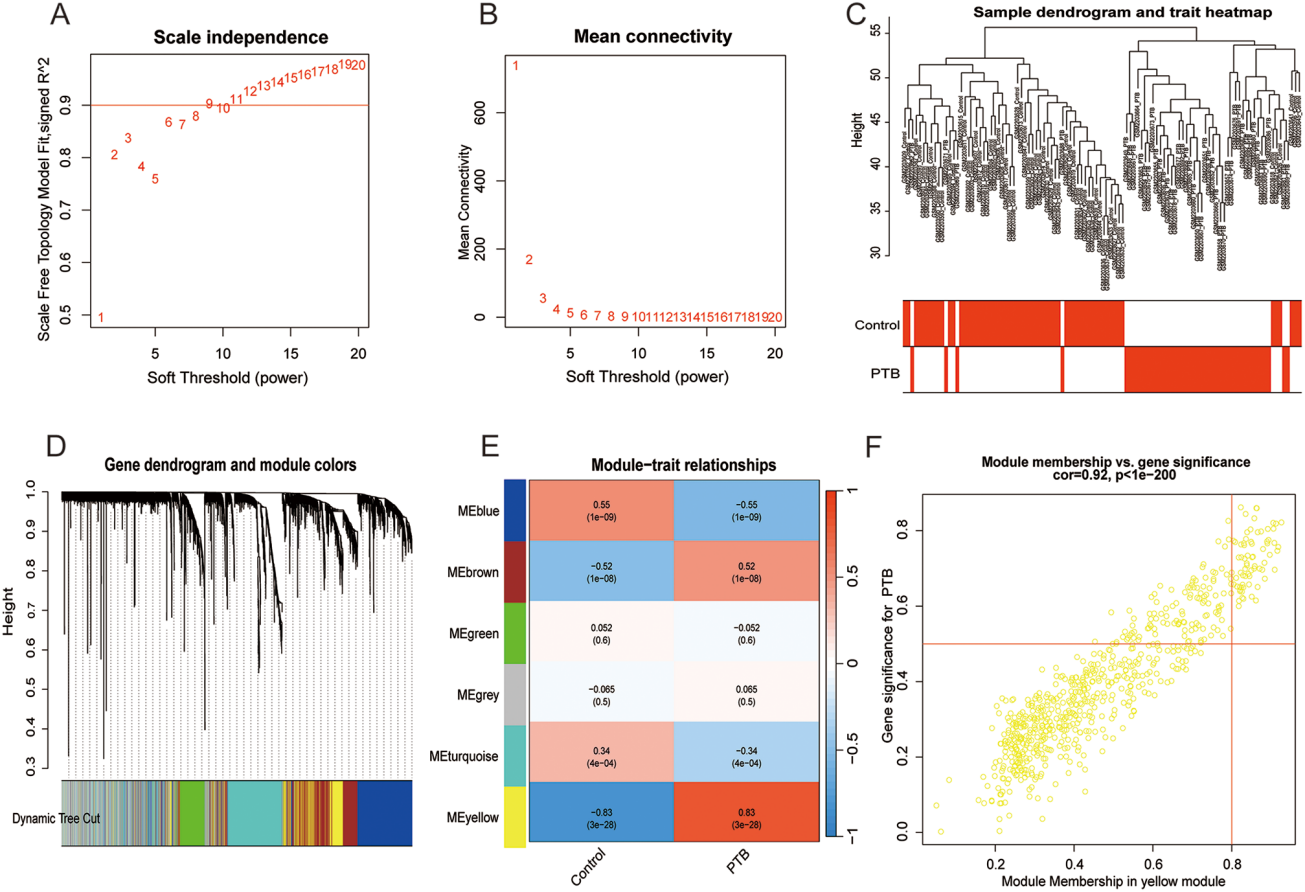
Co-expression network construction and correlation analysis with PTB phenotypes. Coexpression network and correlation analysis with PTB phenotypes. **(A, B)** Scale-free network construction with scale independence and mean connectivity analysis (b = 6). **(C)** Sample clustering tree, with merging of modules with similar expression profiles. **(D)** Module identification: Network dendrogram based on differential measurements and module colors. Each node represents a gene; the vertical axis shows topological differences between genes, and the horizontal axis represents different modules. Each color indicates a module, and the bar width indicates the number of genes in the module. Dynamic Tree Cut was used for the initial clustering, with similar modules merged for the final reconstruction. **(E)** Module-trait correlation: Heatmap showing correlations between modules and TB traits. Cell color indicates correlation strength (deeper red for positive, deeper blue for negative), with the value in each cell indicating the correlation coefficient and the value in brackets showing the *p*-value. **(F)** The scatter plot shows a correlation of 0.92 (*p* < 0.001) between gene significance and module membership in the yellow module, highlighting its strongest association with the PTB phenotype.

Leveraging raw microarray data from the GSE83456 dataset, a gene co-expression network was constructed via the WGCNA package to identify modules associated with PTB and to elucidate potential underlying biological mechanisms. The optimal soft-thresholding power was determined to be 6, based on achieving a scale-free topology fit index of 0.90 coupled with a marked reduction in network connectivity (**Figures 3A, B**). This parameter was subsequently applied to establish the weighted gene co-expression network. Hierarchical clustering of samples with similar expression profiles revealed discrete segregation between the normal control and PTB groups (**Figure 3C**). A topological overlap matrix (TOM) was derived from the adjacency matrix, and gene modules were delineated using the dynamicTreeCut algorithm. Modules exhibiting high similarity were merged according to a threshold of 0.25, resulting in the identification of ten distinct gene modules (**Figure 3D**). Correlation analysis between module eigengenes and clinical traits demonstrated a significant association of the yellow module with PTB, implicating its potentially pivotal role in PTB pathogenesis (**Figure 3E**). Although the brown module exhibited a weak positive relation to PTB and the blue module demonstrated a stronger association with the healthy control group, only the yellow module was ultimately selected for subsequent analysis.

### 3.3 Enrichment analysis of CRGs in PTB

**Figure 4:**
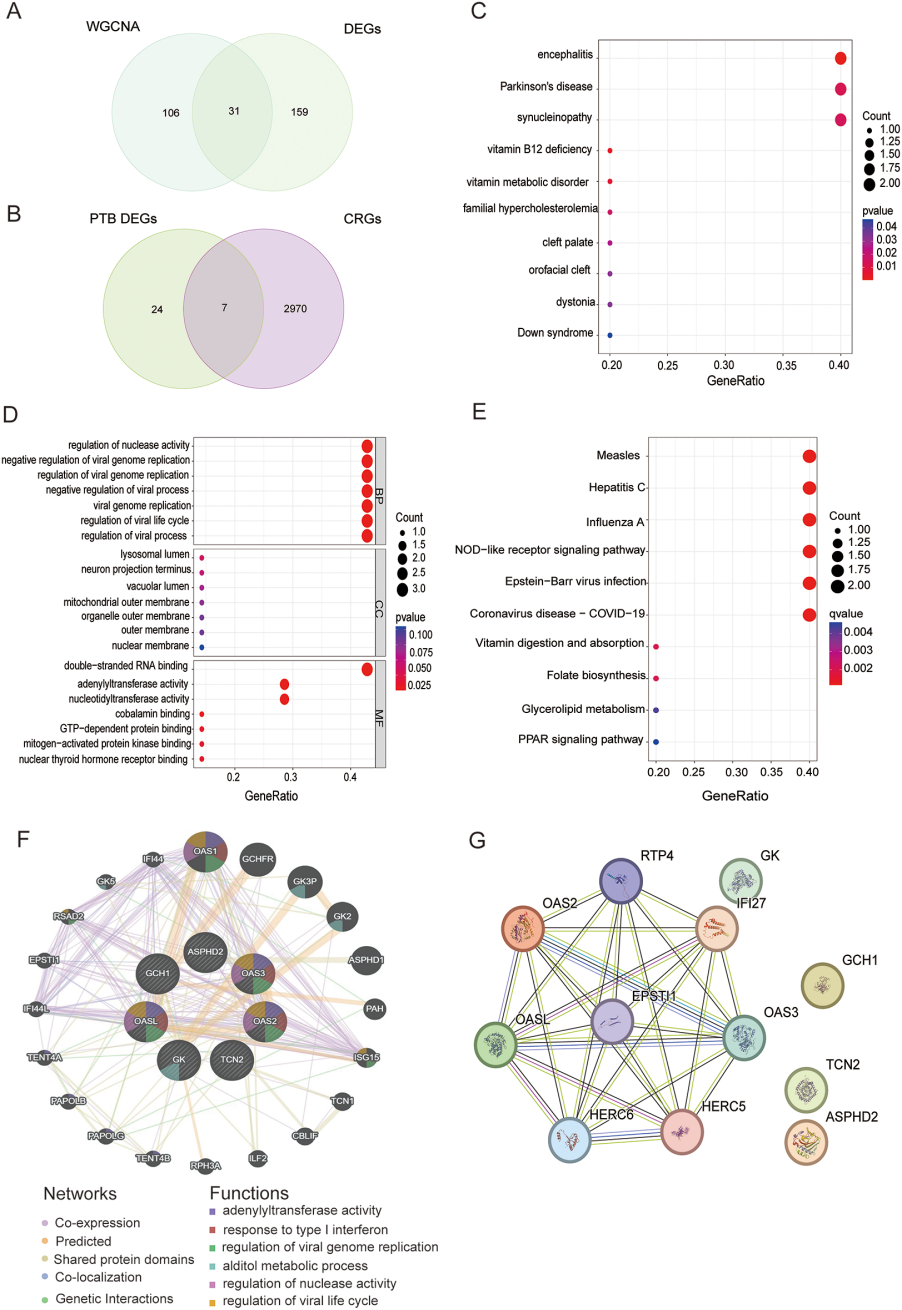
Screening, functional enrichment, and interaction network analysis of PTB CRGs. **(A)** Venn diagram of 137 DEGs significantly associated with PTB identified by WGCNA and 190 DEGs in PTB, yielding 31 PTB intersecting genes. **(B)** Venn diagram of the 31 PTB intersecting genes and 2,977 CRGs, yielding 7 upregulated PTB CRGs: OASL, OAS2, ASPHD2, GK, TCN2, OAS3, and GCH1. **(C)** DO analysis results for PTB CRGs. **(D)** GO analysis of PTB CRGs in BP, CC, and MF. **(E)** KEGG pathway analysis results for PTB CRGs. **(F)** GGI network analysis of PTB CRGs based on the GeneMANIA database, showing the connectivity and functional associations of the identified genes. **(G)** PPI network analysis of PTB CRGs, depicting direct protein interactions and their roles in shared biological pathways.

The intersection of the DEGs significantly linked to PTB identified by WGCNA and 31 PTB DEGs were obtained (**Figure 4A**). These 31 genes were subsequently intersected with CRGs (3), resulting in the identification of seven key PTB CRGs that exhibited significant associations: OASL, OAS2, ASPHD2, GK, TCN2, OAS3, and GCH1. Notably, the expression trends of these seven genes were all upregulated (**Figure 4B**).

Disease Ontology (DO), Gene Ontology (GO), and Kyoto Encyclopedia of Genes and Genomes (KEGG) pathway analyses were performed to further elucidate the functional characteristics of the seven identified PTB-related CRGs. The DO analysis demonstrated that these PTB CRGs were predominantly enriched in diseases and biological mechanisms associated with encephalitis, Parkinson’s disease, and synucleinopathies (**Figure 4C**). Within the GO framework, the biological process (BP) category revealed significant enrichment of PTB CRGs in the regulation of nuclease activity, negative regulation of viral genome replication, and regulation of viral genome replication. In the cellular component (CC) category, these genes were chiefly localized to the lysosomal lumen, neuron projection terminus, and vacuolar lumen. Regarding molecular function (MF), the most significantly enriched terms included double-stranded RNA binding and adenylyl transferase activity (**Figure 4D**). KEGG pathway analysis further indicated that PTB CRGs were primarily involved in pathways related to measles, hepatitis C, influenza A, NOD-like receptor signaling, Epstein-Barr virus infection, and COVID-19 (**Figure 4E**).

### 3.4 Gene interaction network and PPI network

Utilizing the GeneMANIA database, a gene-gene interaction (GGI) network analysis of seven PTB-related CRGs identified the OAS family members (OASL, OAS2, and OAS3) as highly interconnected nodes occupying central positions within the network, thereby highlighting their critical involvement in interferon-mediated antiviral responses and the regulation of viral genome replication. Additionally, GK, GCH1, and TCN2 were implicated in key metabolic pathways, notably glycerol metabolism and vitamin B12 transport. Although ASPHD2 exhibited relatively lower connectivity, its associations with metabolism-related genes suggest a contributory role in specific metabolic processes. Collectively, these findings reinforce the coordinated involvement of PTB CRGs in modulating antiviral immunity and metabolic regulation (**Figure 4F**).

The PPI network consists of 12 nodes and 28 edges, representing the protein products encoded by these genes and their interactions. The nodes are interconnected by edges representing direct PPIs, thereby forming a cohesive network that elucidates their potential involvement in shared biological processes. According to degree centrality, the PTB CRGs are ranked as follows: OASL (6), OAS2 (5), OAS3 (5), GCH1 (3), TCN2 (2), GK (1), and ASPHD2 (1). Notably, OASL, OAS2, and OAS3 exhibit high connectivity, underscoring their pivotal roles in antiviral immune responses. Although GCH1, TCN2, and GK display lower degree values, they are likely integral to metabolic regulation. Specifically, OASL, OAS2, and OAS3 participate in modulating antiviral defenses via the interferon signaling pathway, whereas GCH1, GK, and TCN2 are implicated in metabolic pathways and may contribute to metabolic regulation and related pathologies. Despite their comparatively lower degree values, ASPHD2 and GK may nonetheless play critical roles in specific biological functions (**Figure 4G**).

### 3.5 Identification of key genes associated with disease features using ML algorithms

**Figure 5:**
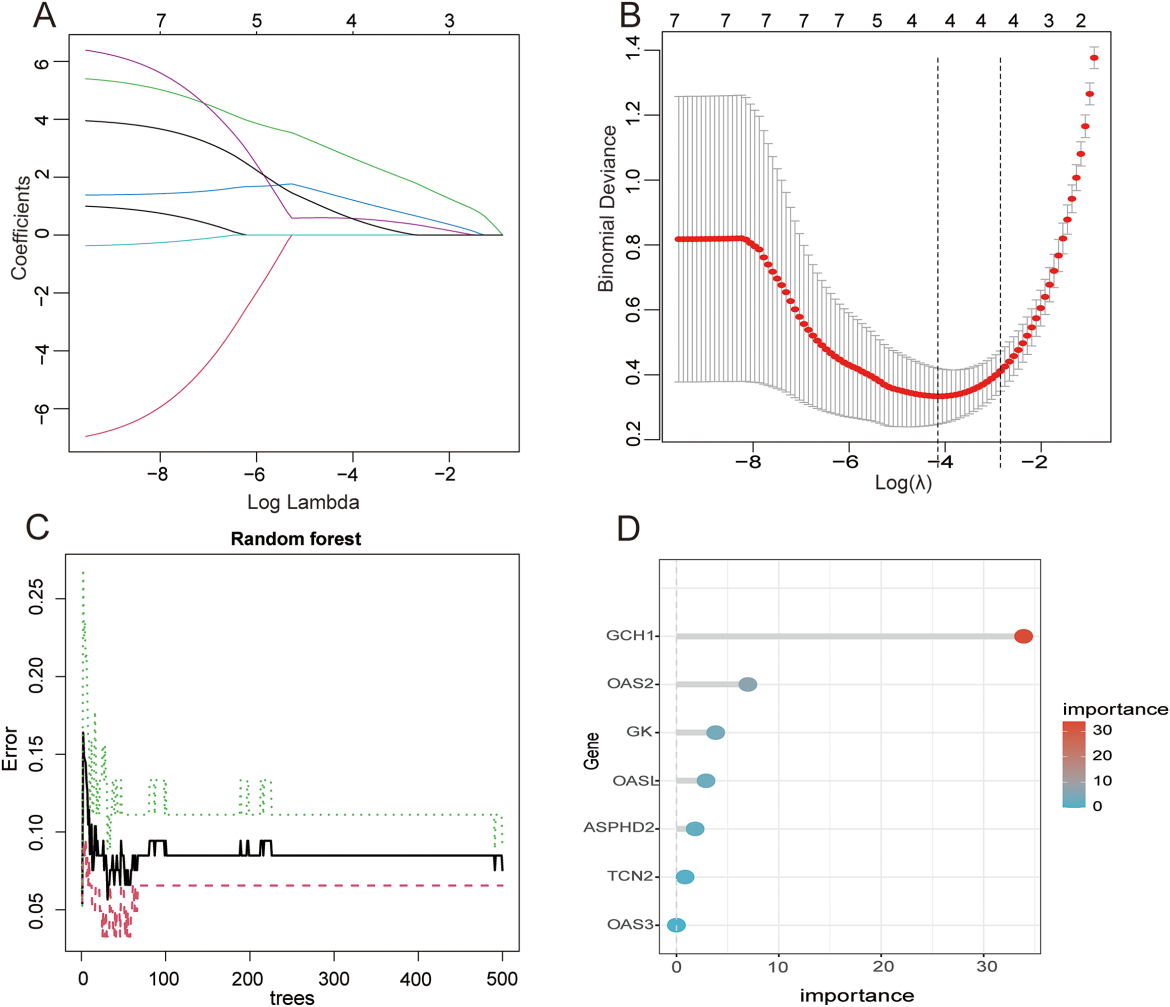
Application analysis of LASSO regression and RF models in the feature selection and prediction of PTB **(A)**Variation of each feature coefficient with the regularization parameter λ in LASSO regression. As λ increases, the feature coefficients gradually decrease and approach zero, indicating that LASSO regression effectively performs feature selection by enhancing the regularization, ultimately resulting in a simplified model. **(B)** Variation of the binomial deviance with the regularization parameter λ in the LASSO regression. Initially, as λ increases, the deviance decreases but subsequently rises, indicating that the optimal λ corresponds to the minimum deviance, balancing model complexity and fitting accuracy. **(C)** Change in the error rate of the RF model with different numbers of trees. As the number of trees rises from 0 to 500, the error rate gradually declines. The error rate tends to stabilize around 300 trees, and a further increase in the number of trees does not significantly improve the error rate. Therefore, 300 trees were selected to optimize performance and save computational resources. **(D)** Gene importance scores in the RF model. The GCH1 gene received the highest score, indicating its significant contribution to the model’s prediction. Other important genes include OAS2, GK, etc. These scores help to identify hub genes in the predictive model and provide guidance for subsequent biological research.

LASSO regression and RandomForest feature selection algorithms were applied to screen out hub genes related to PTB CRGs. The results of LASSO regression showed that ASPHD2, GK, OAS3, and GCH1 were identified as cuproptosis-related hub genes in PTB. While GCH1, OAS2, GK, OASL, ASPHD2, and TCN2 were identified as cuproptosis-related hub genes in PTB using the RandomForest feature selection algorithm. The intersection of these findings revealed three cuproptosis-related hub genes associated with PTB: ASPHD2, GK, and GCH1 (**Figures 5A–D**).

### 3.6 Expression of hub genes in training and validation sets

**Figure 6:**
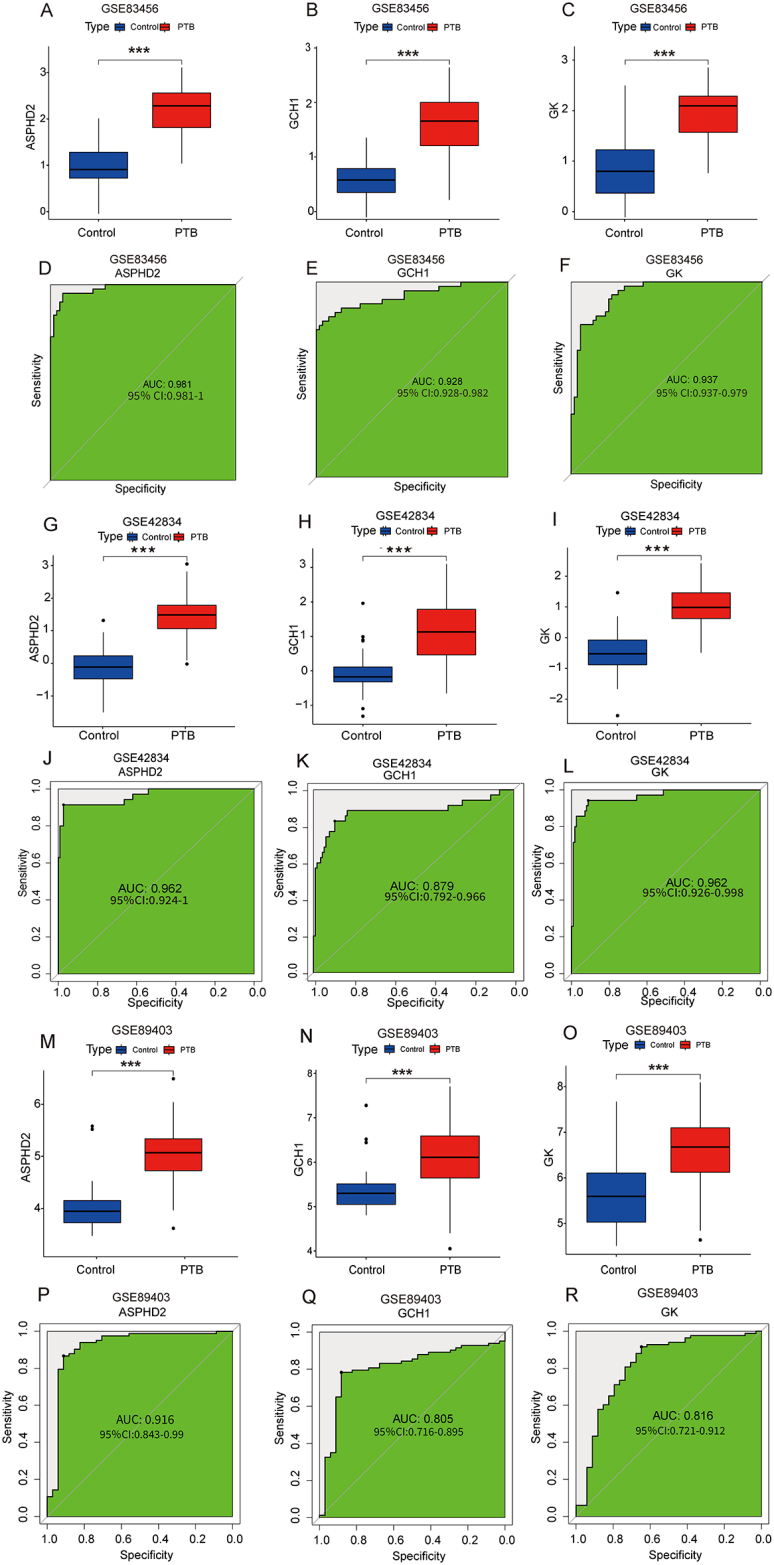
Expression analysis and diagnostic performance of hub genes ASPHD2, GCH1, and GK in PTB patients. **(A–C)** Expression levels of ASPHD2, GCH1, and GK were significantly upregulated in PTB patients in the training set GSE83456 (*p* < 0.001). **(D–F)** ROC curve analysis based on GSE83456 demonstrated excellent diagnostic performance for all three genes: ASPHD2 (AUC = 0.981, 95% CI: 0.981-1.000), GCH1 (AUC = 0.928, 95% CI: 0.928-0.982), and GK (AUC = 0.937, 95% CI: 0.937-0.979). **(G–I)** In the validation set GSE42834, all three genes also showed significantly elevated expression in PTB patients (*p* < 0.001). **(J–L)** ROC analysis in GSE42834 yielded the following results: ASPHD2 (AUC = 0.962, 95% CI: 0.924-1.000), GCH1 (AUC = 0.879, 95% CI: 0.792-0.966), and GK (AUC = 0.962, 95% CI: 0.926-0.998). **(M–O)** In the validation set GSE89403, the expression levels of all three genes remained significantly elevated in PTB patients (*p* < 0.001). **(P–R)** ROC analysis of GSE89403 confirmed robust diagnostic performance: ASPHD2 (AUC = 0.916, 95% CI: 0.843-0.990), GCH1 (AUC = 0.805, 95% CI: 0.716-0.895), and GK (AUC = 0.816, 95% CI: 0.721-0.912). An AUC > 0.9 indicates excellent diagnostic accuracy; 0.8-0.9 indicates good accuracy; 0.7-0.8 is considered acceptable; and an AUC < 0.7 reflects poor diagnostic performance. ***p < 0.001.

In the training dataset GSE83456, the expression levels of the three hub genes, ASPHD2, GCH1, and GK, were significantly elevated in PTB patients in contrast to healthy controls (all *p* < 0.001). The ROC curve, a widely recognized tool for evaluating diagnostic performance, employs the AUC as a key metric. An AUC greater than 0.9 indicates excellent diagnostic accuracy; values between 0.8 and 0.9 denote good accuracy; 0.7 to 0.8 is considered acceptable; whereas an AUC below 0.7 signifies poor diagnostic performance. In the GSE83456 cohort, the AUC values for ASPHD2, GCH1, and GK were 0.981, 0.928, and 0.937, respectively, each demonstrating excellent diagnostic efficacy (**Figures 6A–F**). In the validation dataset GSE42834, all three genes exhibited significant upregulation in PTB patients (*p* < 0.001), with ROC analysis revealing AUCs of 0.962 for ASPHD2, 0.879 for GCH1, and 0.962 for GK (**Figures 6G–L**), indicative of good to excellent diagnostic performance. Likewise, in the independent validation dataset GSE89403, these genes remained markedly upregulated in PTB patients (*p* < 0.001), with corresponding AUCs of 0.916, 0.805, and 0.816 for ASPHD2, GCH1, and GK, respectively (**Figures 6M–R**). Collectively, these findings affirm the robustness and diagnostic utility of these hub genes across diverse cohorts and heterogeneous experimental platforms.

### 3.7 Construction and validation of diagnostic prediction models

**Figure 7:**
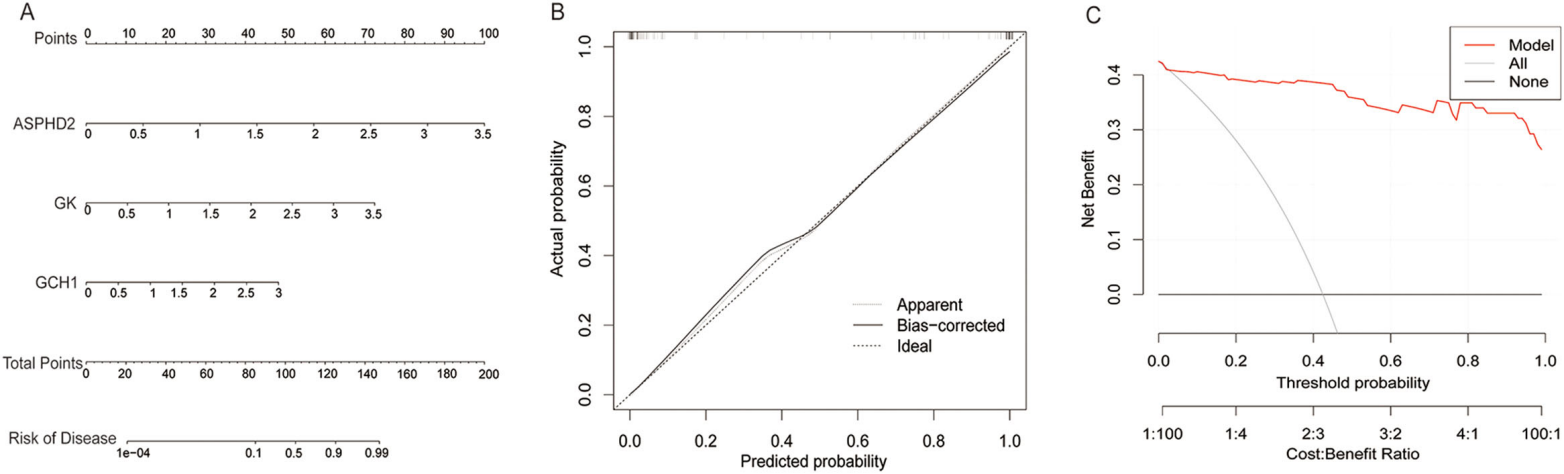
Construction and clinical evaluation of the PTB risk prediction model. **(A)** Nomogram for predicting PTB risk based on the expression levels of ASPHD2, GK, and GCH1 genes. **(B)** Calibration curve comparing the predicted probability with the actual probability. The predicted probability is close to the ideal line (diagonal line), indicating the excellent calibration performance of the model. **(C)** Decision curve analysis shows that the model provides a high and stable net benefit within a threshold probability range of 0 to 0.6, demonstrating its significant clinical application value in predicting PTB risk.

To evaluate the prognostic significance of cuproptosis-related hub genes in the onset and progression of PTB, a nomogram model was developed incorporating ASPHD2, GK, and GCH1. This model quantifies disease risk by integrating the expression levels and corresponding scores of these hub genes to yield a composite predictive score (**Figure 7A**). The calibration curve demonstrated excellent concordance between predicted and observed probabilities along the ideal 45-degree line, underscoring the robust calibration performance of the nomogram (**Figure 7B**). Moreover, decision curve analysis (DCA) revealed that within a threshold probability range of 0 to 0.6, the model exhibits substantial clinical utility in risk stratification for PTB (**Figure 7C**).

### 3.8 Cell type identification and distribution estimation tool (CIBERSORT)

**Figure 8:**
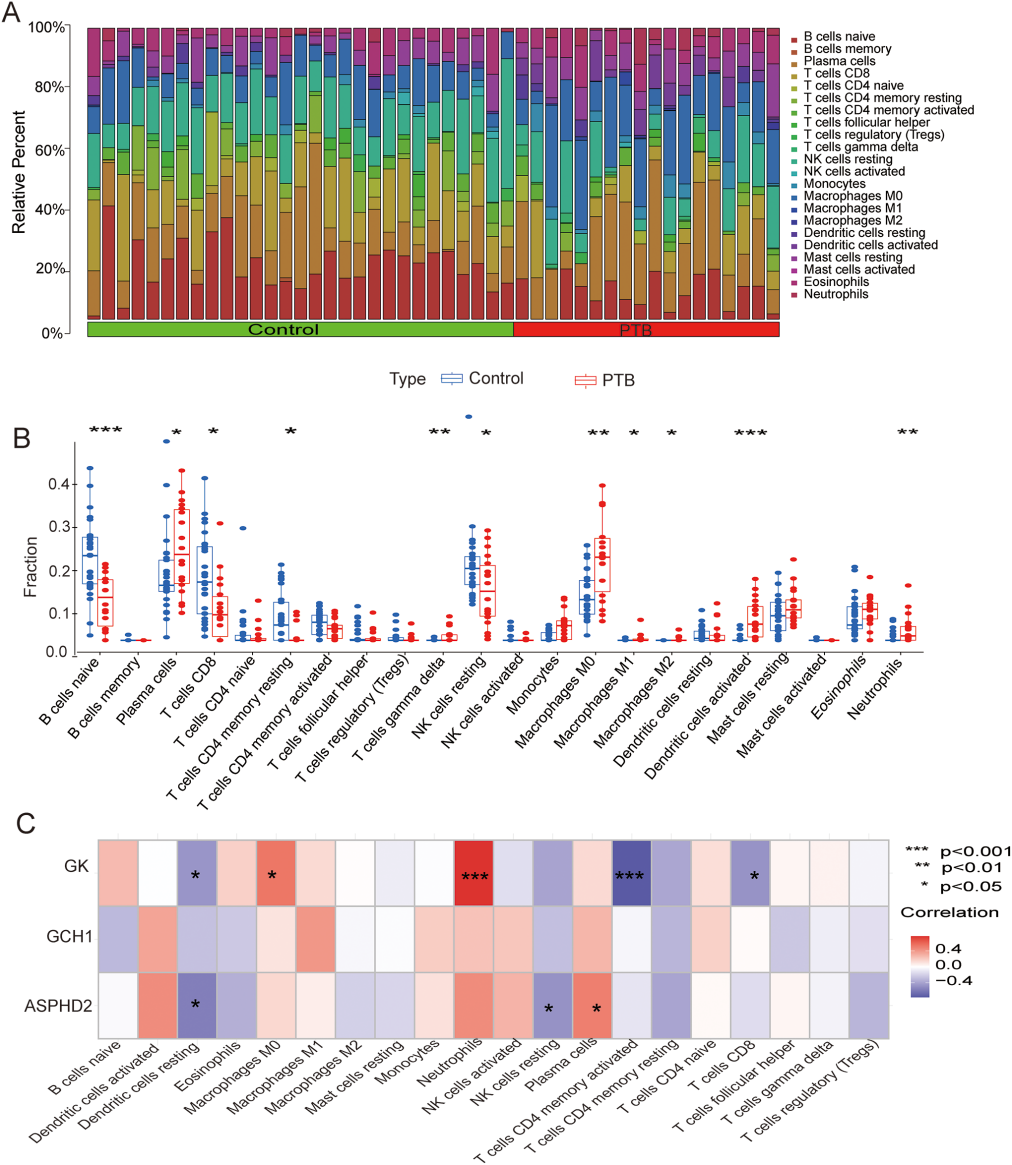
CIBERSORT analysis of PTB patients and its correlation with cuproptosis-related hub genes in PTB patients. **(A)** Analysis of immune cell infiltration in PTB patients and the NC group. CIBERSORT analysis revealed significant differences in the relative abundance of 22 immune infiltrating cell types between the two groups. **(B)** Scatter plot showing the differences in immune cell infiltration between the PTB group and the NC group. **(C)** Correlation analysis between cuproptosis hub genes and infiltrating immune cells. *p < 0.05, **p < 0.01, ***p < 0.001.

To elucidate the immunological disparities between PTB patients and healthy controls, our study employed CIBERSORT to quantify the relative proportions of 22 immune cell subsets in both cohorts. The analysis revealed significant differences in the abundance of these infiltrating immune cell populations between the two groups (**Figure 8A**). Within the PTB cohort, notable alterations in immune cell infiltration were observed. Specifically, plasma cells, gamma delta T cells, macrophage subsets (M0, M1, and M2), activated dendritic cells, and neutrophils exhibited a marked increase, implicating their potential pivotal role in the immunopathogenesis of PTB. Conversely, naïve B cells, CD8^+^ T cells, resting memory CD4^+^ T cells, and resting natural killer (NK) cells were significantly diminished, suggesting their possible suppression in the immune regulatory milieu of PTB (**Figure 8B**). Furthermore, correlation analysis between immune infiltrates and cuproptosis-related hub genes demonstrated that GK expression was significantly positively correlated with neutrophils and M0 macrophages (*p* < 0.001 and *p* < 0.05, respectively) while showing a significant negative correlation with activated memory CD4^+^ T cells, resting dendritic cells, and CD8^+^ T cells (*p* < 0.001 and *p* < 0.05, respectively). The ASPHD2 gene was significantly positively correlated with plasma cells (*p* < 0.05), and significantly negatively related to dendritic cells resting and NK cells resting (*p* < 0.05) (**Figure 8C**).

### 3.9 ssGSEA

**Figure 9:**
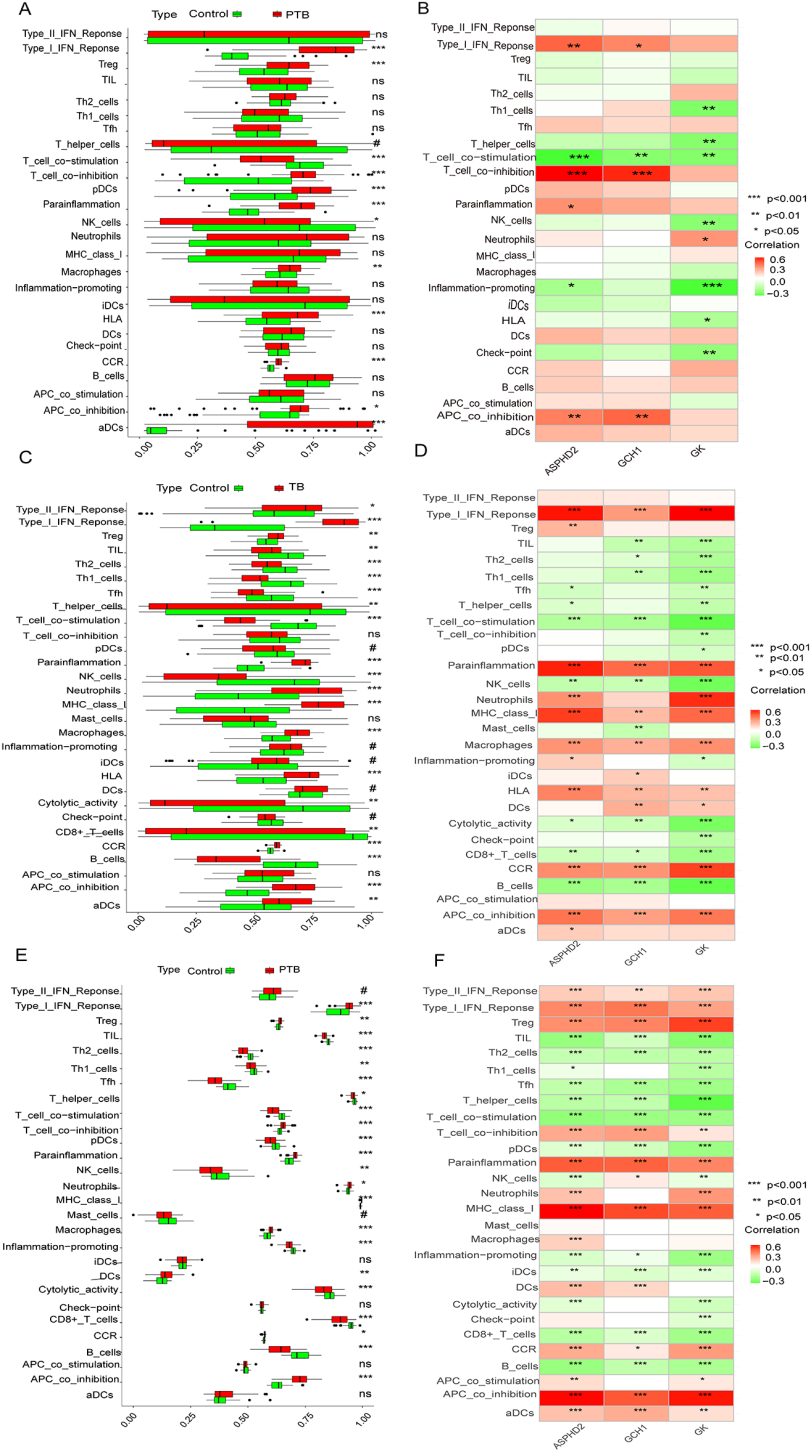
Differences in immune functions and cell types between PTB patients and normal controls, and correlation analysis with hub genes and immune features. **(A, B)** Analysis results from the training set GSE83456.**(A)** Significant differences in various immune functions and cell infiltration between the PTB and NC groups. **(B)** Correlation analysis between hub genes and immune features. **(C, D)** Analysis results from the validation set GSE42834.**(C)** Significant differences in various immune functions and cell infiltration between the PTB and NC groups. **(D)** Correlation analysis between hub genes and immune features. **(E, F)** Analysis results from the validation set GSE89403.**(E)** Significant differences in various immune functions and cell infiltration between the PTB and NC groups. **(F)** Correlation analysis between hub genes and immune features.*Note: The X-axis represents ssGSEA scores (0-1) or hub genes, and the Y-axis represents immune features. Green represents NC and red represents PTB. *p < 0.05, **p < 0.01, ***p < 0.001. #*p* ≥ 0.05; p < 0.2, near-significant results. ns, not significant.

The ssGSEA analysis showed significant differences in various immune functions and immune cell types between the PTB group and the normal control group. In the PTB group, there was a significant increase in type I interferon response (*p* < 0.001), regulatory T cells (Treg, *p* < 0.001), T cell co-inhibition (*p* < 0.001), plasmacytoid dendritic cells (pDCs, *p* < 0.001), parainflammation (*p* < 0.001), macrophages (*p* < 0.01), human leukocyte antigen (HLA, *p* < 0.001), chemokine receptors (CCR, *p* < 0.001), antigen-presenting cell co-inhibition (APC co-inhibition, *p* < 0.05), and activated dendritic cells (aDCs, *p* < 0.001). Meanwhile, T cell co-stimulation (*p* < 0.001) and NK cells (*p* < 0.05) were significantly decreased in the PTB group. These results indicate that PTB patients have significant immune dysfunction, presenting a state of both innate immune activation and immune suppression (**Figure 9A**).

The ssGSEA analysis demonstrated significant alterations in diverse immune functions and immune cell populations between the PTB cohort and the healthy control group. Specifically, the PTB group exhibited marked elevations in type I interferon response (p < 0.001), regulatory T cells (Tregs, p < 0.001), T cell co-inhibition (p < 0.001), plasmacytoid dendritic cells (pDCs, p < 0.001), parainflammation (p < 0.001), macrophages (p < 0.01), human leukocyte antigen (HLA) expression (p < 0.001), chemokine receptor (CCR) activity (p < 0.001), antigen-presenting cell co-inhibition (APC co-inhibition, p < 0.05), and activated dendritic cells (aDCs, p < 0.001). Conversely, significant reductions were observed in T-cell co-stimulation (p < 0.001) and natural killer (NK) cells (p < 0.05) within the PTB group. These findings collectively indicate profound immune dysregulation in PTB patients, characterized by concurrent activation of innate immunity alongside mechanisms of immune suppression (**Figure 9A**).

Further, ssGSEA revealed significant associations between the expression of ASPHD2, GCH1, and GK genes and multiple immune functions and cell types, with correlations spanning six, four, and eight categories, respectively. The ASPHD2 gene showed significant positive correlations with type I interferon response, T cell co-inhibition, APC co-inhibition, and parainflammation, and significant negative correlations with T cell co-stimulation and pro-inflammatory activities. Similarly, GCH1 expression was positively correlated with type I interferon response, T cell co-inhibition, and APC co-inhibition, but negatively correlated with T cell co-stimulation. The GK gene exhibited a significant positive correlation with neutrophil abundance while demonstrating significant negative correlations with Th1 cells, T helper cells, T cell co-stimulation, NK cells, pro-inflammatory functions, HLA expression, and immune checkpoint molecules (**Figure 9B**). Collectively, ASPHD2, GCH1, and GK are pivotal regulators of immune activation and suppression, contributing to immune homeostasis and the prevention of excessive immune responses. Notably, GK appears to exert broad inhibitory effects on multiple immune functions, potentially facilitating immune evasion or modulation of inflammatory processes.

To further unravel the immune-related characteristics of the hub genes, ssGSEA was conducted across two independent validation datasets (GSE42834 and GSE89403). The findings closely mirrored those observed in the training cohort. In both validation datasets, PTB patients exhibited markedly heightened type I interferon (IFN) responses, increased T cell co-inhibition, and enhanced macrophage infiltration, accompanied by a notable attenuation of T cell co-stimulation and natural killer (NK) cell activity. These results collectively delineate a reproducible pattern of immune dysregulation. Moreover, the associations between the hub genes and immune-related functions remained robust and consistent across the datasets. Specifically, GK demonstrated sustained negative correlations with pro-inflammatory signaling pathways, T helper type 1 (Th1) cells, general helper T cells, and NK cells in both validation sets, underscoring its potential involvement in immunosuppressive processes and immune homeostasis. Likewise, ASHPD2 and GCH1 retained their characteristic immunological correlation profiles, both exhibiting significant positive associations with type I IFN response, parainflammatory activity, and antigen-presenting cell (APC) co-inhibition. The foregoing findings underscore the consistent immunomodulatory roles of the three hub genes across both training and validation cohorts, reflecting a distinctive immunological landscape in PTB characterized by the simultaneous presence of innate immune activation and immunosuppressive states. This cross-cohort concordance reinforces the robustness and biological significance of our conclusions (**Figures 9C–F**).

### 3.10 Validation of cuproptosis-related hub genes in PTB

**Figure 10:**
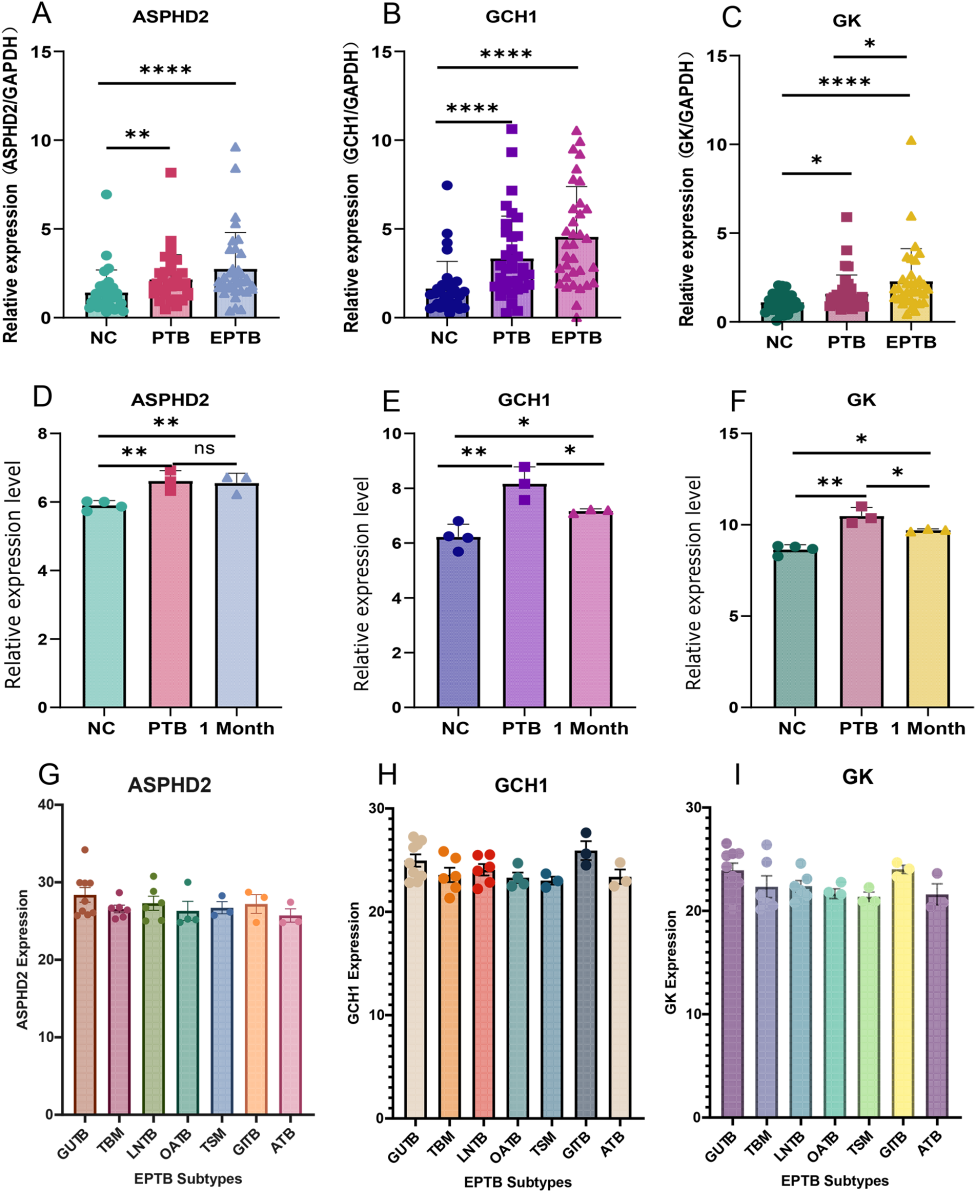
Validation and expression analysis of cuproptosis-related hub genes in PTB. **(A–C)** The relative expression levels of ASPHD2, GCH1, and GK genes in normal individuals, PTB patients, and EPTB patients were analyzed by RT-qPCR. **(D–F)** Relative expression levels of ASPHD2, GCH1, and GK genes in NC, PTB patients before treatment, and PTB patients after 1 month of anti-TB treatment, based on gene chip expression profiles. **(G–I)** Expression levels of ASPHD2, GCH1, and GK genes in different subtypes of EPTB. Patients were categorized based on the main affected sites: genitourinary TB (GUTB, n = 9), tuberculous meningitis (TBM, n = 6), lymph node TB (LNTB, n = 6), osteoarticular TB (OATB, n = 4), tuberculous spinal meningitis (TSM, n = 3), gastrointestinal TB (GITB, n = 3), and abdominal TB (ATB, n = 3). No statistically significant differences in gene expression were observed among these EPTB subtypes. X-axis: group classification. Y-axis: relative gene expression levels. *p < 0.05, **p < 0.01, ****p < 0.0001, ns, not significant.

PBMCs isolated from blood samples of 31 NC, 35 PTB, and 34 EPTB patients were analyzed for differential expression of hub genes by RT-qPCR. The basic clinical characteristics of the patients are summarized in **Table 2**, with no statistically significant differences in age and gender distribution between the two groups of patients. The results showed that the expression levels of ASPHD2, GCH1, and GK genes were significantly higher in both the PTB and EPTB groups compared to the normal control group (*p* < 0.05) (**Figures 10A–C**).

Gene chip expression profiling analysis was performed on samples from 4 NC subjects and 3 PTB patients before and after 1 month of anti-TB treatment. The results showed that the expression levels of ASPHD2, GCH1, and GK genes were significantly higher in the PTB group than in the NC group (*p* < 0.05). After 1 month of standard chemotherapy, the expression levels of GCH1 and GK genes, although not returning to normal levels, were significantly reduced (*p* < 0.05), whereas no significant changes in ASPHD2 gene expression were observed. The expression levels of GCH1 and GK genes were found to correlate with disease improvement, which suggested that GCH1 and GK possibly serve as potential biomarkers for early therapeutic monitoring in PTB (**Figures 10D–F**). To further elucidate the expression patterns in EPTB, a subgroup analysis was performed based on the principal sites of infection. Patients with EPTB were classified into the following subtypes: genitourinary TB (GUTB, n= 9), tuberculous meningitis (TBM, n= 6), lymph node TB (LNTB, n= 6), osteoarticular TB (OATB, n= 4), tuberculous spinal meningitis (TSM, n= 3), gastrointestinal TB (GITB, n= 3), and abdominal TB (ATB, n= 3). No statistically significant differences in the expression of ASPHD2, GCH1, or GK were observed among the various EPTB subtypes (**Figures 10G–I**).

**Figure 11:**
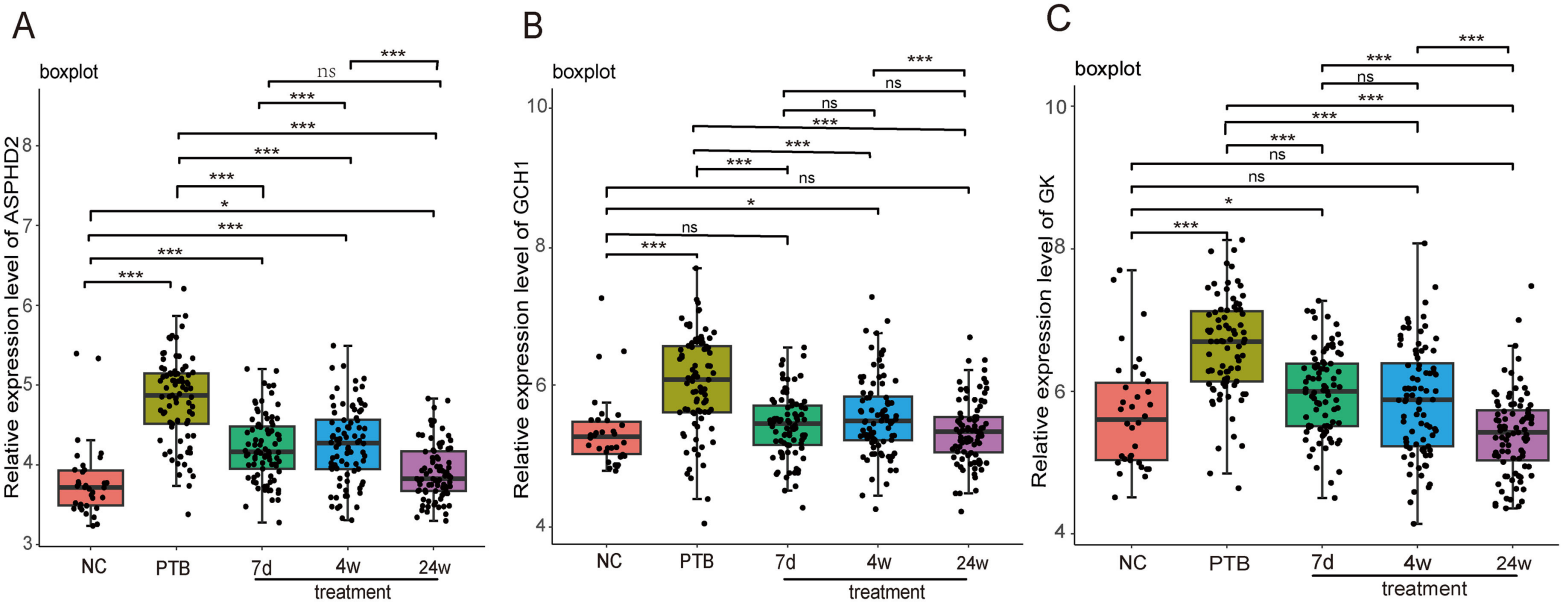
Dynamic analysis of the expression of PTB cuproptosis-related hub genes in PTB patients during treatment in the GSE89403 dataset. X-axis: Grouping information, including normal control, pulmonary tuberculosis (PTB), after 7 days of treatment (7d treatment), after 4 weeks of treatment (4w treatment), and after 24 weeks of treatment (24w treatment). Y axis: Relative expression level. *p < 0.05, ***p < 0.001, ns stands for not significant.

To further validate the potential of the PTB cuproptosis-related hub genes as therapeutic monitoring biomarkers, the GSE89403 dataset was selected for verification, including 34 NC samples, 83 PTB samples before treatment, and 85, 84, and 87 PTB samples after 7 d, 4 w, and 24 w treatment, respectively. The analysis results showed that the expression levels of ASPHD2, GCH1, and GK genes in the pre-treatment PTB group were significantly higher than those in the NC group (*p* < 0.001). Among these, GCH1 was the most sensitive to treatment, with its expression level decreasing significantly and approaching that of the NC group after 7 d of treatment (*p* < 0.001). After 24 weeks of treatment, GCH1 expression showed no significant difference from the NC group (*p* > 0.05). GK showed a similar trend, with a notable decrease in its expression level after 7 d treatment (*p* < 0.001), but there was still a significant difference compared to the NC group (*p* < 0.05). After 4 weeks of treatment, its expression level approached that of the NC group (*p* > 0.05), and the difference with the NC group was further reduced after 24 weeks of treatment. The difference with the NC group was further reduced after 24 weeks of treatment. ASPHD2 expression decreased significantly after treatment (*p* < 0.001). Although it had not returned to normal levels after 24 weeks of treatment (*p* < 0.05), its expression level continued to decrease as treatment progressed. In summary, the cuproptosis-related hub genes GCH1, GK, and ASPHD2 in PTB can serve as potential biomarkers for monitoring therapeutic efficacy in the early, intermediate, and late stages of PTB (**Figures 11A–C**).

**Figure 12:**
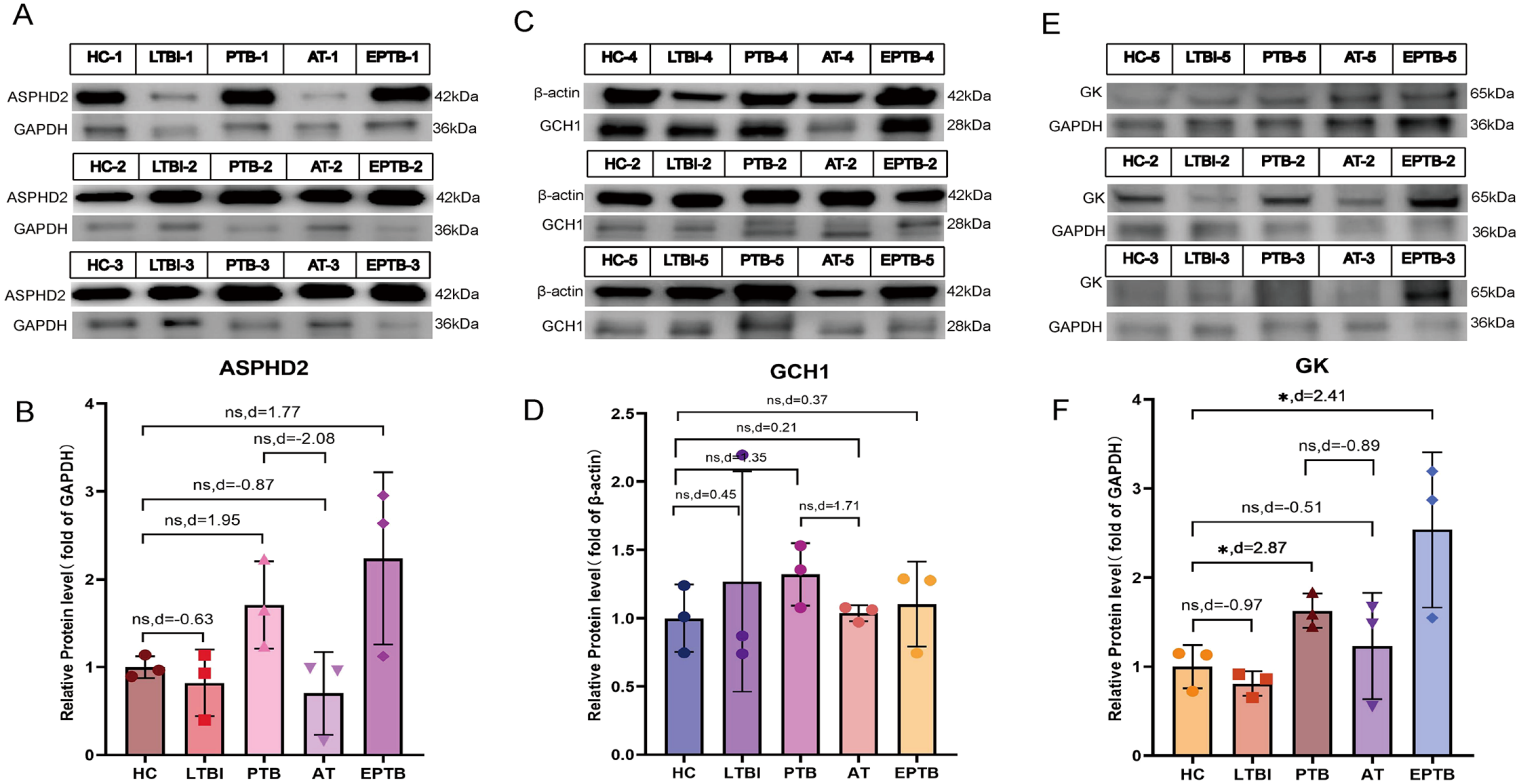
WB analysis of ASPHD2, GCH1, and GK protein expression in PBMCs across five clinical groups. **(A, C, E)** Representative WB bands for ASPHD2, GCH1, and GK in healthy controls (HC), latent TB infection (LTBI), active pulmonary TB (PTB), PTB after one month of anti-TB treatment (AT), and extrapulmonary TB (EPTB), with molecular weights (kDa) indicated. **(B, D, F)** Quantification of relative protein expression levels. Data are presented as mean ± SD with individual data points overlaid. Effect sizes were assessed using Cohen’s *d* to compare group differences.

To further substantiate the findings derived from RT-qPCR and microarray analyses, WB assays were carried out using PBMC samples obtained from five clinical cohorts, to validate the protein expression levels of the candidate cuproptosis-related hub genes ASPHD2, GCH1, and GK. The cohorts comprised HC, LTBI, PTB, AT, and EPTB cohorts. Representative WB bands corresponding to ASPHD2, GCH1, and GK are presented in **Figures 12A, C, E**, respectively, with the associated molecular weights (kDa) duly annotated.

In comparison to the HC group, ASPHD2 protein expression was markedly elevated in both PTB and EPTB groups. Although conventional statistical tests did not achieve significance (P > 0.05), effect size analysis employing Cohen’s d demonstrated pronounced differences, with values of d = 1.95 for PTB versus HC and d = 1.77 for EPTB versus HC, indicative of a marked upregulation of ASPHD2 in ATB. Furthermore, ASPHD2 expression was markedly diminished in the after-treatment (AT) group relative to PTB (d = −2.08), suggesting its potential utility as a biomarker for therapeutic response (**Figure 12B**). Expression of GCH1 was likewise significantly elevated in the PTB cohort (d = 1.35) and declined following treatment (AT vs. PTB, d = −1.71), approximating baseline levels observed in HC (AT vs. HC, d = 0.21). The disparity between the EPTB and HC groups was comparatively modest (d = 0.37), implying limited biological relevance (**Figure 12D**).

GK protein levels were markedly elevated in both the PTB group (effect size, d = 2.87) and the EPTB group (d = 2.41) relative to the HC group (*p* < 0.05), indicating very large effect sizes. Following treatment, expression levels in the AT group decreased (d = −0.51), although they did not fully revert to baseline values (**Figure 12F**). In the LTBI group, protein expression exhibited greater variability and did not differ significantly from that of the HC group (P > 0.05). Notably, the expression of all three proteins in both the PTB and EPTB groups consistently surpassed levels observed in the LTBI group, reflecting a pattern congruent with the overall comparisons against the HC cohort.

## 4 Discussion

TB, among the leading infectious diseases causing death worldwide, is a major public health threat, particularly in developing countries (14). Approximately 83% of new TB cases are PTB (15). Currently, PTB diagnosis and treatment in clinical practice face multiple challenges, as the pathogenic mechanisms of PTB remain elusive. Recently, cuproptosis has been identified as a novel form of cell death harmful to the host. This study used ML to identify the hub genes of PTB-related CRGs, analyzed their immunological characteristics, and, for the first time, investigated their potential value in the diagnosis and therapeutic monitoring of PTB.

In 2022, Tsvetkov P et al. identified a set of genes associated with cuproptosis by a genome-wide CRISPR screening (3). In 2023, Li S et al. discovered that 11 (NFE2L2, NLRP3, ATP7B, SLC31A1, MTF1, DLD, LIAS, LIPT1, DLAT, GLS, and DBT) of these genes were associated with TB-related cuproptosis (8). Chen L et al. found that 9 (MTF1, NFE2L2, NLRP3, FDX1, LIPT1, PDHB, GLS, DBT, and DLST) of these genes were related with cuproptosis in pediatric ATB (9). This study found for the first time that three hub genes (ASPHD2, GK, and GCH1) were associated with cuproptosis in PTB. These hub genes were upregulated in PTB patients and significantly higher than those in the NC group. ROC curve analysis showed that these hub genes had some diagnostic value for PTB, and the nomogram model constructed based on ASPHD2, GK, and GCH1 had a high clinical application value in predicting the risk of PTB.

Validation using a separate dataset, RT-qPCR, and gene expression profiling sequencing showed that ASPHD2, GCH1, and GK had the same expression trend in both the PTB and EPTB groups. GSEA analysis of DEGs in PTB showed that upregulated genes were mainly enriched in pathways related to pathogen infection and immune response, and KEGG analysis of PTB CRGs also showed significant enrichment in pathways related to pathogen infection and antigen recognition. These results confirm that 3 hub genes possibly play an important role in the pathogenesis and progression of TB. Further validation at the protein level via WB analysis substantiated these findings, revealing consistently elevated expression of ASPHD2, GCH1, and GK in PBMCs derived from PTB patients and EPTB. Although conventional statistical testing did not yield significant differences (P > 0.05), Cohen’s d effect size analysis demonstrated large magnitude differences for ASPHD2 (PTB vs. healthy controls [HC], d = 1.95; EPTB vs. HC, d = 1.77), indicative of considerable biological relevance. Notably, ASPHD2 expression exhibited a marked decline following one month of anti-tuberculosis treatment (d = −2.08), underscoring its potential utility as a dynamic biomarker for monitoring therapeutic response in tuberculosis. Similarly, GCH1 expression was significantly elevated in the PTB cohort (d = 1.35) and showed a pronounced reduction post-treatment (d = −1.71), approaching baseline levels comparable to those observed in healthy controls (d = 0.21). Conversely, the differential expression of GCH1 between the EPTB and HC groups was modest (d = 0.37), suggesting a relatively limited involvement of this gene in extrapulmonary disease. In contrast, GK expression was markedly upregulated in both PTB (d = 2.87) and EPTB (d = 2.41) with very large effect sizes. Although expression levels did not fully normalize following therapy, a discernible reduction was evident in the AT group (d = −0.51). Collectively, these multi-tiered validation data robustly support the involvement of ASPHD2, GCH1, and GK in tuberculosis pathogenesis and immune activation, highlighting their potential as protein-level biomarkers for the assessment of treatment response. In particular, the favorable expression kinetics of ASPHD2 and GCH1 during therapy suggest their clinical applicability for monitoring therapeutic efficacy, especially in the early to intermediate stages of PTB.

Further validation using GEO datasets and gene expression profiling sequencing revealed that GCH1, GK, and ASPHD2 genes were sensitive to therapeutic efficacy during PTB treatment. GCH1 approached normal levels after 7 days of treatment, GK approached normal levels after 4 weeks of treatment, and ASPHD2 expression gradually decreased with the extension of treatment. For the first time, GCH1, GK, and ASPHD2 were identified as potential biomarkers for monitoring the efficacy of PTB treatment in early, middle, and late stages. Our future studies will further evaluate their clinical value in monitoring treatment response.

GTP cyclohydrolase 1 (GCH1) is the rate-limiting enzyme in the tetrahydrobiopterin (BH4) synthesis pathway, catalyzing the conversion of GTP to 7,8-dihydrobiopterin triphosphate (D-PTP). It is a key regulatory point in the synthesis of BH4 (16, 17). BH4 is an essential cofactor for several enzymes, such as nitric oxide synthase (NOS), and plays a variety of critical biological roles in the body, particularly in the immune system. BH4 regulates the production of the reactive oxygen species (ROS) and stabilizes the formation of the inducible nitric oxide synthase (iNOS) dimer. INOS regulates many host innate immune cells and adaptive immune responses via nitric oxide (NO) to control or eliminate MTB and exert anti-inflammatory effects that enhance host defense function (18). Furthermore, BH4 plays an antioxidant role, maintaining redox balance, scavenging ROS, and protecting cells from oxidative stress-induced damage (16). GCH1 affects NO production by regulating BH4 synthesis, thereby modulating immune cell function. It plays a role in a variety of immune-related diseases, such as infectious diseases, autoimmune diseases, and cancer, by influencing disease progression and immune evasion mechanisms. GCH1 also contributes to the immune response and inflammatory response in TB (19). Based on whole blood gene expression profiling, Qian Z et al (20) found that GCH1 expression levels were significantly higher in TB patients compared to healthy controls, people with latent TB infection, pneumonia, and cancer patients. Furthermore, anti-TB chemotherapy led to a reduction in GCH1 expression levels, which is consistent with our findings. McNeill E et al. (19) showed a significant positive correlation between GCH1 gene expression in human PBMCs and increased BCG colony-forming units (CFUs). However, the Gch1fl/flTie2cre (Gch1 knockout/BH4-deficient) mice infected with BCG showed weight gain, no significant pathological changes in the lung and spleen, but a significant decrease in the lung and spleen CFUs. This study further demonstrated a significant upregulation of GCH1 expression in PTB patients (**Figure 10**). Collectively, these findings indicate that the host mounts a response to MTB infection by enhancing BH4 synthesis, underscoring the pivotal role of the GCH1 gene in the pathogenesis of tuberculosis. Moreover, attenuation of GCH1 expression appears to augment macrophage-mediated control of mycobacterial infection. Although no studies have directly proved the relationship between GCH1 gene variants and susceptibility or resistance to TB, GCH1 knockout (Gch1^-/-^) macrophages are deficient in BH4, resulting in impaired nitric oxide production. NO plays a critical role in the ability of macrophages to control MTB infection (19, 21). Gene expression analysis indicates that GCH1-deficient macrophages control MTB infection by altering inflammatory responses, lysosomal function, cell survival, and cellular metabolism (19). Therefore, GCH1 possibly serves as a biomarker for the diagnosis and efficacy evaluation of active PTB, as well as a potential therapeutic target.

Glycerol kinase (GK) encodes glycerol kinase, which catalyzes the phosphorylation of glycerol to glycerol-3-phosphate (G3P) with ATP, representing a key step in glycerol metabolism (22). GK is mainly expressed in the liver and kidney, where it provides glycerol-3-phosphate as an intermediate for glycolysis, glycogen synthesis, gluconeogenesis, and triglyceride synthesis, thus closely influencing cellular energy metabolism, fatty acid metabolism, glucose metabolism, and other physiological processes (23). GK regulates glycerol metabolism and affects the energy metabolic pathways of immune cells such as macrophages, thereby influencing immune cell survival, activation, function, and inflammatory responses (24). GK acts as a co-regulator of nuclear receptor subfamily 4 group A1 (NR4A1) and is involved in the regulation of hepatic glucose metabolism by interacting with NR4A1 proteins. Overexpression of GK inhibits NR4A1’s regulation of hepatic gluconeogenesis target genes and glucose homeostasis both *in vitro* and *in vivo* (22, 23), thereby leading to excessive cellular carbon consumption and glucose lipotoxicity, disrupting intracellular energy balance, and ultimately inducing apoptosis. In addition, GK1b has been shown to activate key apoptotic proteins (25–27). No studies have been reported on GK gene variation or expression and susceptibility or resistance to TB. This study found for the first time that the expression of the CRG-related gene GK was significantly upregulated in PTB patients and showed a significant decrease during treatment. The increase in GK expression possibly induces cell apoptosis, cuproptosis, and other forms of cell death in PTB patients, promotes glycolysis and lipid synthesis, and provides a carbon source for MTB, which are associated with MTB infection-induced metabolic reprogramming of host cells, and play a role in the pathogenesis of TB. The decrease in GK expression after treatment suggests restoration of energy metabolism and disease remission in patients. Future studies will further elucidate the key role of GK in cuproptosis pathways and host immune regulation, as well as explore the reliability of GK as a therapeutic efficacy assessment and therapeutic target.

Aspartate β-hydroxylase domain-containing 2 (ASPHD2) is a gene encoding a protein containing an aspartate β-hydroxylase domain. It is mainly located at the cell membrane and has α-ketoglutarate (α-KG)-dependent dioxygenase activity and metal ion binding activity. ASPHD2 is involved in redox reactions and peptide chain modifications, regulates aspartate metabolism, and influences the balance of amino acid metabolism (28, 29). α-KG is a key intermediate in the TCA cycle, which not only plays an essential role in cellular metabolism but is also involved in the regulation of various biological processes such as epigenetic changes and immune responses (30–32). Aspartate metabolism possibly be related to cellular antioxidant capacity and inflammatory responses. Recent studies have shown that the ASPHD2 gene is involved in the regulation of monkeypox virus infection and septicaemic melioidosis (33, 34). However, no study has been reported on the association of ASPHD2 gene variation or expression with susceptibility or resistance to TB. This study showed for the first time that ASPHD2 expression increased in PTB patients and decreased after treatment, suggesting that ASPHD2 may be involved in the pathogenesis of TB by regulating aspartate metabolism and redox state to activate cuproptosis-related pathways. Further investigations are necessitated to elucidate its precise mechanism of action in PTB, including its potential interactions with the TCA cycle and immune response-related signaling pathways, as well as its impact on the functional dynamics of immune cells in PTB patients, to further assess its viability as a therapeutic target. Recent research has shown that the abundance of infiltrating immune cells in TB patients has undergone significant changes. Li S et al. reported a significant increase in monocytes, M0, M1, and M2 macrophages, activated dendritic cells, eosinophils, and neutrophils in TB patients (8). Chen L et al. found a significant increase in neutrophils, dendritic cells, and monocytes in children with ATB (9). This study showed that plasma cells, T-cell gamma delta, M0 macrophages, M1 macrophages, M2 macrophages, activated dendritic cells, and neutrophils were significantly increased in PTB patients. These findings suggest that the activation of innate immune cells (such as M1 macrophages, monocytes, and dendritic cells) plays a critical role in host defense against TB while enhancing antigen presentation to induce adaptive immune responses. On the other hand, the immunosuppressive effect of M2 macrophages possibly helps to prevent tissue damage, but possibly also leads to persistent chronic inflammation (35, 36). The increased number of plasma cells reflects the activation of the humoral immune response, potentially leading to a shift from Th1-type to Th2-type immunity (35). In addition, the extensive infiltration of neutrophils possibly contributes to inflammatory tissue damage and exacerbates disease (37). Studies have also shown a significant reduction in CD8^+^ T cells, resting memory CD4^+^ T cells, and follicular helper T cells in TB patients (4). In children with ATB, a decrease in infiltration of CD8^+^ memory T cells, activated CD8^+^ T cells, CD4^+^ memory T cells, activated CD4^+^ T cells, and B cells has been observed (5) However, this study showed a significant decrease in naive B cells, CD8 T cells, resting memory CD4 T cells, and resting NK cells in PTB patients. These results highlight the impact of MTB infection on the host immune environment and the immunopathological features of PTB, confirming that the occurrence and development of PTB are associated with lymphocyte suppression, in particular with significant reductions in effector T cells (CD8^+^ T cells and CD4^+^ T cells) involved in anti-TB cellular immunity, and B cells participating in accessory anti-TB humoral immunity. Furthermore, NK cells, which play a anti-TB cytotoxic role in both innate and adaptive immunity, were also significantly reduced, suggesting that PTB is primarily characterized by impaired adaptive immunity, immune suppression, or exhaustion of effector T cells (35). Association analysis between the CRGs ASPHD2, GK, and GCH1 and immune cell infiltration showed that the GK gene was significantly positively correlated with neutrophils and M0 macrophages, while negatively correlated with activated CD4 T cells, dendritic cells, and CD8 T cells, indicating that GK is mainly closely related to the immunopathology and adaptive immune deficiency in PTB (35, 37). ASPHD2 gene was positively correlated with plasma cells and negatively correlated with dendritic cells and NK cells, suggesting that ASPHD2 mainly promotes the antibody-mediated function of plasma cells and is closely related to the inhibition of antigen presentation and NK cell activity (35). The GCH1 gene showed no significant correlation with changes in immune cell infiltration. The association between the CRGs GK and ASPHD2 and changes in immune cell infiltration further suggests that cuproptosis induced by their high expression possibly plays a significant role in the pathogenesis of PTB.

Further analysis using ssGSEA revealed significant changes in immune function and immune cell composition in PTB patients, with significant increases in chemokine receptors (CCR), human leukocyte antigens (HLA), macrophages, and activated dendritic cells (aDCs), indicating enhanced immune cell recruitment and innate immunity. Conversely, a significant increase in antigen-presenting cell co-inhibition (APC co-inhibition) and T-cell co-inhibition, together with a significant reduction in T-cell co-stimulation and NK cells, suggested suppression of adaptive immunity. In addition, the significant increase in type I interferon response, regulatory T cells (Treg), plasmacytoid dendritic cells (pDCs), and parainflammation suggested the formation of an immunosuppressive microenvironment that possibly contributes to immune evasion and chronic inflammation in PTB (35, 38). The findings from the ssGSEA analysis were consistent with the results from the CIBERSORT analysis, reflecting an immune dysregulation in PTB patients, characterized by the coexistence of enhanced innate immunity and immune suppression. This immunopathological feature possibly be related to host-pathogen interactions and disease progression. ASPHD2, GCH1, and GK genes play pivotal roles in modulating immune activation and suppression. Among them, the ASPHD2 and GCH1 genes were significantly positively correlated with immune function suppression and immune evasion, such as type I IFN response, T cell co-inhibition, and APC co-inhibition, while the GK gene showed a significant positive correlation with neutrophils and a negative correlation with adaptive immune function. This suggests that GK, together with ASPHD2 and GCH1, possibly be involved in the decline of anti-TB immunity and immune-inflammatory damage (35, 37).

This study has limitations, which warrant further refinement in subsequent investigations: (1) Expansion of sample size: While the present cohort offers a degree of representativeness, it remains insufficient to capture the full spectrum of clinical manifestations. Future studies will aim to enlarge the sample pool to encompass tuberculosis (TB) patients and healthy controls from a broader array of geographic regions and ethnic backgrounds, thereby enabling a more comprehensive validation of the involvement of ASPHD2, GK, and GCH1 in the pathogenesis of TB. (2) Functional validation: Flow cytometry and single-cell transcriptomic sequencing will be employed to characterize T cell subsets in the peripheral blood or tissue samples of PTB patients, to identify subtle alterations in the expression of key genes within MTB-specific T cells. The functional relevance of these genes in PTB pathogenesis will be elucidated by modulating T-cell subpopulations and assessing the effects of gene knockdown or overexpression on MTB proliferation in both *in vitro* and *in vivo* models. (3) Investigation of genetic variation: Given the complexity of TB pathophysiology, particularly concerning drug resistance and host genetic variability, targeted sequencing will be conducted to explore the association between gene mutations and TB susceptibility or resistance. Further research is essential to elucidate the mechanistic implications of these genetic variants for TB pathogenesis and diagnostic biomarker development, ultimately contributing to the advancement of personalized diagnostic and therapeutic strategies.

## 5 Conclusion

In conclusion, this study is the first to identify three hub genes, ASPHD2, GCH1, and GK, implicated in the pathogenesis of PTB through integrated screening using LASSO and RF models. These genes exhibited significantly elevated expression levels in PTB patients relative to healthy controls, with expression markedly diminished following anti-tuberculosis therapy. This dynamic trend was further validated at the protein level by WB analysis, reinforcing the consistency between transcriptomic and proteomic data. WB. The marked alterations in immune cell infiltration and immune function observed in the PTB population may be closely associated with cuproptosis mediated by GK, ASPHD2, and GCH1. This process appears to promote the activation of innate immune cells, including macrophages, monocytes, dendritic cells, and neutrophils, while concurrently suppressing the function of adaptive immune cells. These findings suggest that the ASPHD2, GK, and GCH1 genes may be indirectly implicated in the pathogenesis of PTB through their influence on pathways including amino acid metabolism, glucose metabolism, and nitric oxide synthesis. Therefore, the CRG hub genes ASPHD2, GCH1, and GK possibly serve as potential biomarkers for the diagnosis and therapeutic monitoring of active PTB, as well as potential therapeutic targets.

## Data Availability

The datasets presented in this study can be found in online repositories. The names of the repository/repositories and accession number(s) can be found below: https://www.ncbi.nlm.nih.gov/, GSE83456, GSE42834, GSE54992, GSE89403.
